# Site-Specific Introduction of Negative Charges on the Protein Surface for Improving Global Functions of Recombinant Fetal Hemoglobin

**DOI:** 10.3389/fmolb.2021.649007

**Published:** 2021-03-30

**Authors:** Karin Kettisen, Cedric Dicko, Emanuel Smeds, Leif Bülow

**Affiliations:** ^1^Division of Pure and Applied Biochemistry, Department of Chemistry, Lund University, Lund, Sweden; ^2^Division of Infection Medicine, Department of Clinical Sciences, Lund University, Lund, Sweden

**Keywords:** protein engineering, fetal hemoglobin, protein surface charge, DNA cleavage, small-angle X-ray scattering, plasma half-life

## Abstract

Due to its compatible oxygen-transporting abilities, hemoglobin (Hb) is a protein of interest in the development of artificial oxygen therapeutics. Despite continuous formulation attempts, extracellular Hb solution often exhibits undesirable reactions when applied *in vivo*. Therefore, protein engineering is frequently used to examine alternative ways of controlling the unwanted reactions linked to cell-free Hb solutions. In this study, three mutants of human fetal hemoglobin (HbF) are evaluated; single mutants αA12D and αA19D, and a double mutant αA12D/A19D. These variants were obtained by site-directed mutagenesis and recombinant production in *E. coli*, and carry negative charges on the surface of the α-subunit at the designated mutation sites. Through characterization of the mutant proteins, we found that the substitutions affected the protein in several ways. As expected, the isoelectric points (pIs) were lowered, from 7.1 (wild-type) down to 6.6 (double mutant), which influenced the anion exchange chromatographic procedures by shifting conditions toward higher conductivity for protein elution. The biological and physiological properties of HbF could be improved by these small modifications on the protein surface. The DNA cleavage rate associated with native HbF could be reduced by 55%. In addition, the negatively charged HbF mutant had an extended circulation time when examined in a mouse model using top load Hb additions. At the same time, the mutations did not affect the overall structural integrity of the HbF molecule, as determined by small-angle X-ray scattering. In combination with circular dichroism and thermal stability, modest structural shifts imposed by the mutations could possibly be related to changes in secondary structure or reorganization. Such local deformations were too minor to be determined within the resolution of the structural data; and overall, unchanged oxidation and heme loss kinetics support the conclusion that the mutations did not adversely affect the basic structural properties of Hb. We confirm the value of adding negatively charged residues onto the surface of the protein to improve the global functions of recombinant Hb.

## Introduction

In humans, efficient oxygen transport and delivery are provided by human hemoglobin (Hb), a protein contained in high concentrations inside the red blood cells. In the event of hemolysis, Hb leaks into the vascular compartment and exhibits oxidative toxicity, nitric oxide scavenging, and causes heme-associated inflammatory responses. Under normal conditions, these adverse effects are carefully controlled by efficient endogenous scavengers and Hb clearing pathways, including hemopexin, haptoglobin, and CD163 (Rother et al., 2005; Schaer and Alayash, 2010).

In the field of oxygen therapeutics, many formulations of hemoglobin-based oxygen carriers (HBOCs) are based on the administration of extracellular Hb solutions. When applying significant amounts of extracellular Hb, it effectively mimics the events of hemolysis and exhibits toxic effects. Extracellular Hb used for these applications must therefore be modified to avoid such severe side reactions. The majority of extracellular HBOCs are based on Hb obtained from human or bovine sources, and different formulations include conjugation to non-protein macromolecules, intramolecular crosslinking, and polymerization (Alayash, 2014). However, so far, no HBOC candidate has been approved for human use by key governmental agencies such as the Food and Drug Administration in the United States and the European Medicines Agency in the European Union. HBOCs that have entered into clinical trials have often shown adverse events such as hypertension, acute renal failure, and even death (Buehler et al., 2010), and these highly unwanted events have prompted more comprehensive studies of the underlying molecular reactions.

Studies of recombinant Hb are aiming to clarify the relations of extra- and intramolecular events preceding Hb-mediated toxicity. With recombinant production, any Hb molecule—natural, mutated, or modified—can be produced for functional studies. In analogy to all vertebrate hemoglobins, human fetal hemoglobin (HbF) is a tetramer and consists of two α-subunits and two γ-subunits (α_2_γ_2_). Compared to adult hemoglobin (HbA, α_2_β_2_), HbF has several differentiating qualities, such as a higher alkali denaturation resistance (Perutz, 1974), higher solubility (Frier and Perutz, 1977), lower tetramer-dimer dissociation constant (Yagami et al., 2002), and higher affinity for oxygen in the presence of 2,3-diphosphoglycerate (Bauer et al., 1968; Bunn and Briehl, 1970). In previous work, we reported a mutant of HbF, which showed decreased oxidative reactivity compared to wild-type HbF (wtHbF) (Kettisen et al., 2018). This mutant is part of an effort to examine rational site-directed mutagenesis to mitigate adverse oxidative reactivity of future Hb-based products. Related mutants with reported moderated oxidative reactivity are for example engineered mutants focusing on tyrosine residues at various locations of the Hb molecule (Cooper et al., 2019), and a naturally occurring variant involving an aspartate in place of lysine in the β subunit (Strader et al., 2017).

Another strategy is to camouflage Hb from the surrounding environment by decreasing interactions with other biomolecules in the extracellular compartments, e.g., by connecting the Hb proteins to serum albumin (Tomita et al., 2013). Alternatively, the molecular weights of the HBOC molecules can be increased. Previous and ongoing HBOC formulations therefore utilize chemical conjugation to attach for example polyethylene glycol (PEG) (Conover et al., 1999; Manjula et al., 2005; Winslow, 2008), and/or employ chemical cross-linking of the globin chains (Guillochon et al., 1981; Chatterjee et al., 1986; Gould et al., 1998). The motif is to increase the molecular size or stabilize the tetrameric conformation of the Hb protein to reduce the rate of plasma clearance, as the tetramer is less easily removed from circulation through glomerular filtration in the kidneys (Bunn et al., 1969).

In the interest of contributing to the Hb engineering strategies at disposal, we have investigated the option of lowering the pI and increasing the net charge of the HbF molecule itself. Without attaching additional modifying molecules or utilizing tags or genetic linking, we have in this study substituted alanine residues for aspartate residues in the α-subunit and examined the impact on properties such as functionality, oxidative and thermal stability, structure, and plasma retention. We focus on mutations on the α-subunit partly due to being the subunit with the higher pI, and partly due to the well-known solubility issues of this part of the Hb protein. Unaccompanied α-subunits are prone to precipitate, and *in vivo* synthesis of Hb involves a molecular chaperone, α-hemoglobin stabilizing protein (AHSP), which binds α-subunits before pairing up with β-subunits (Weiss et al., 2005). In heterologous host cells such chaperones are not present, and the difficulty of unaccompanied α-subunit expression is a recognized problem. One way to address this issue has been to co-express the α-subunit and AHSP in the recombinant host to increase the soluble yield of the α-subunit (Vasseur-Godbillon et al., 2006). However, if the α-subunit can be engineered to become more soluble on its own, the production yield of recombinant Hb could be improved without the need for a chaperone. Negatively charged mutations may have other positive effects as well. Mrabet et al. and Adachi et al. have previously shown enhanced dimer and tetramer formation of natural and recombinant mutants of HbA when up to two additional negative charges were present on the β-subunit (Mrabet et al., 1986; Adachi et al., 1998). The tetrameric form of Hb is essential for function and is also more oxidatively stable than the dimers (Griffon et al., 1998).

In this study, we have included two α-subunit mutation sites to examine the potential of this strategy for HbF. The chosen mutations were; (1) substitution of alanine to aspartate at position 12, and (2) substitution of alanine to aspartate at position 19. We compared the three mutants of HbF; single mutants αA12D and αA19D, and double mutant αA12D/A19D; to wtHbF. The mutation αA12D (Hb Paris) occurs as a natural mutant in several cases around the world and is reported to have normal stability (Rosa et al., 1966; Trincao et al., 1968; Dash and Huisman, 1988). The αA19D mutation (Hb Kurosh) has also been reported (Rahbar et al., 1976), as well as a similar mutation at this position with substitution into glutamic acid (Hb Tashikuergan, αA19E) (Houjun et al., 1984), and both these mutant variants display normal stability with no clinical symptoms. These natural occurrences show that mutations into aspartate at these sites most likely will not adversely disturb the normal functionality and stability of the protein, and thus might be valuable target sites for lowering the pI and increasing surface net charge without adverse effects on the HbF protein.

## Materials and Methods

### Recombinant HbF Production

**Figure 1:**
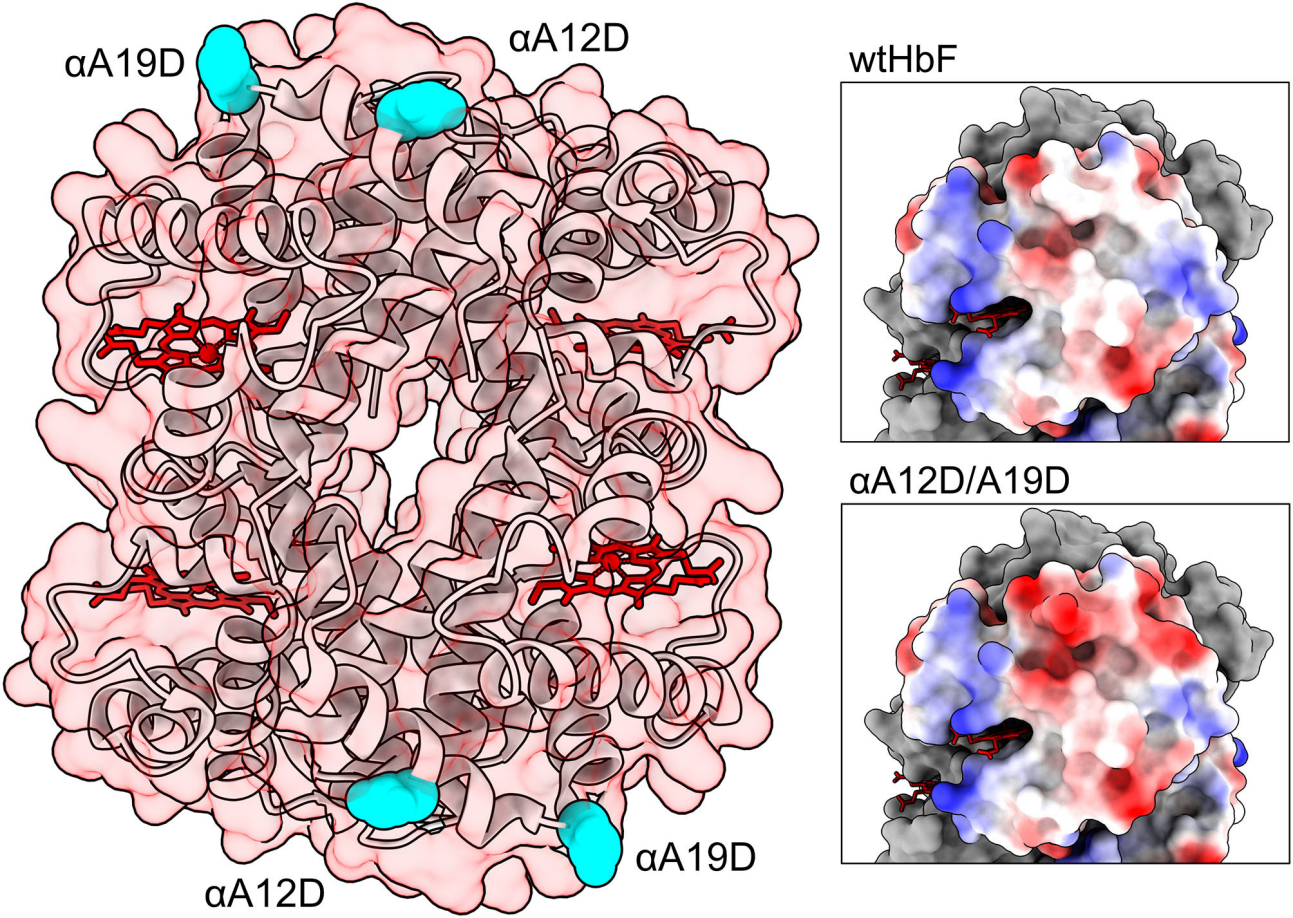
Structure of HbF (PDB code: 1FDH; Frier and Perutz, 1977). To the left the entire protein structure is shown as ribbons, with a transparent red-tinted surface, and heme groups are colored in red. Mutation sites are depicted as cyan spheres. Panels to the right show the surface of the α-subunit colored by electrostatic potential (red for negative, blue for positive, and white for neutral). The upper right panel show unmodified wild-type HbF while the lower panel show the double mutated structure αA12D/A19D. The figures were created in the UCSF Chimera and ChimeraX molecular visualization programs (Pettersen et al., 2004; Goddard et al., 2018). Mutations were introduced in the model with the swapaa command in the Chimera software.

Site-directed mutagenesis of the α-subunit gene, previously inserted into a pETDuet-1 plasmid containing both the α- and γ-subunit genes, was performed as described before (Ratanasopa et al., 2016; Kettisen et al., 2018). The locations of the mutations are displayed in Figure 1. The three mutants were formed by introducing negatively charged aspartate in place of hydrophobic alanine—creating two single mutants and a double mutant harboring both mutations. Primers were ordered from Integrated DNA Technologies (Germany). Sequence confirmation and transformation of mutated plasmids into *Escherichia coli* BL21 (DE3), as well as expression and purification of wtHbF and all three mutants, were performed as previously described (Kettisen et al., 2018), with a few modifications. In short, 600 ml Terrific Broth cultures in 2 L Erlenmeyer shake flasks, containing 0.3 mM δ-aminolevulinic acid (Sigma-Aldrich), were induced at OD 0.1 with 0.1 mM isopropyl β-D-1-thiogalactopyranoside (IPTG, Saveen & Werner) and cultivated at 30°C overnight. Carbon monoxide gas (CO) was bubbled into the medium at the end of cultivation, and the cells were subjected to washing with buffer, disruption by sonication, clarification by centrifugation, and filtration to remove the remaining cell debris. The HbF proteins were then extracted from the *E. coli* lysates by performing two steps of ion-exchange chromatography on an ÄKTA Avant 25 system (GE Healthcare), resulting in >95% purity of the monomer band as determined by densitometric analysis with sodium dodecyl sulfate-polyacrylamide gel electrophoresis (SDS-PAGE). The concentrated, purified Hb samples (Hb-CO form) were rapidly frozen with liquid nitrogen and stored at −80°C until use. All Hb concentrations were calculated on a heme-basis throughout the experiments.

### Basic Functionality of HbF Mutants

The HbF mutants were examined by spectrophotometric analysis on a Cary 60 UV-Vis spectrophotometer (Agilent Technologies), in different ligand-bound and heme iron oxidative forms of hemoglobin, i.e., Hb-CO, Hb-O_2_, Hb (Fe^2+^), and Hb (Fe^3+^). Hb-CO spectrum was recorded after bubbling the Hb samples with CO gas. Hb-O_2_ spectrum was achieved by exposing the Hb to a steady stream of pure O_2_ gas under a light source to promote photolysis of the heme-CO complex. Unliganded ferrous Hb (Fe^2+^) spectrum was obtained by adding sodium dithionite (Sigma-Aldrich) to Hb-O_2_. In contrast, ferric Hb (Fe^3+^) spectrum was recorded after adding potassium ferricyanide (K_3_[Fe(CN)_6_], Sigma-Aldrich), incubating on ice while exposing the sample to intense light, and then passing through a Sephadex G-25 desalting column (GE Healthcare) to remove excess oxidizing reagent. Spectra were compared to reported data (Antonini and Brunori, 1971; Meng and Alayash, 2017). The isoelectric points (pIs) were examined by loading the Hb proteins onto a Novex® pH 3–10 IEF gel (Invitrogen) with IEF Standards (Bio-Rad). The electrophoresis was run in an XCell SureLock™ Mini-Cell (Invitrogen) in three phases; 100 V for 1 h, 200 V for 1 h, and 500 V for 30 min. Samples were prepared with IEF sample buffer (Invitrogen), and 10 μg of each HbF sample was loaded per well. The gel was fixed in 12% trichloroacetic acid for 30 min and then stained with Coomassie blue to visualize the protein bands. Autoxidation rates of 10 μM Hb-O_2_ to Hb (Fe^3+^) were examined by recording the spectral change every 30 min at 20°C for 24 h; and the heme loss were monitored at 37°C for 8 h with the heme scavenger apomyoglobin H64Y/V67F (apoMb) (Hargrove et al., 1994); according to procedures described previously (Kettisen et al., 2018). A component analysis in the spectrum range 350–700 nm was used to obtain the heme loss rates (Singh et al., 2020).

### DNA Cleavage

All Hb variants were converted to the ferric form according to the K_3_[Fe(CN)_6_]-procedure described in the previous section. The DNA cleavage activity was examined as described in a previous study (Chakane et al., 2017), with some modifications. Purified pUC18 plasmid DNA 3 ng/μl was incubated at 37°C with 75 μM Hb, in 20 mM phosphate buffer pH 7.2 for 12 h. Samples were removed once every hour up to 6 h, and the last sample was extracted at 12 h. The samples were analyzed on 1% agarose gels and the band corresponding to the supercoiled form of pUC18 was quantified densitometrically with the Image Lab™ software from Bio-Rad Laboratories. The data were fitted to an exponential decay equation with Solver in Excel (Microsoft).

### Thermostability Using Nanodsf

The stability was studied by monitoring thermal denaturation with differential scanning fluorimetry (DSF) in a Prometheus NT.48 instrument (Nano Temper Technologies). O_2_-bound ferrous (Fe^2+^) and ferric (Fe^3+^) samples were prepared at 3 mg/ml and loaded into capillaries. The intrinsic fluorescence intensity ratio between 350 and 330 nm was measured over a temperature ramp of 7°C/min from 20 to 95°C. The data (*n* = 6) was processed in PR.therm Control v.2.04 to determine the onset and inflection temperatures for each sample.

### Small-Angle X-ray Scattering and Data Analysis

All SAXS experiments were carried out on the SWING beamline (https://www.synchrotron-soleil.fr/en/beamlines/swing) at the synchrotron SOLEIL (Saint-Aubin, France) in size exclusion mode, using HPLC system Agilent capillary 1200 system equipped with a SEC-3 150 Å column from Agilent. Concentrated and purified HbF proteins were diluted in 100 μl of 10 mM Tris-HCl buffer at pH 7.45. For each sample, a volume of 40 μl was injected and eluted at 0.3 ml/min in 10 mM Tris buffer at pH 7.45 through a thermalized quartz capillary of 1.5 mm diameter and 10 μm wall thickness, inserted within a vacuum chamber (David and Pérez, 2009). Individual 1 s time frames were collected at 15°C. To account for the interparticle interactions, solutions at protein concentrations in the range of 1–0.25 mg/ml were measured. At the beginning of the elution curve, 180 frames were taken as the buffer to be subtracted from the data. The 2D scattering patterns were reduced into 1D intensities and selected for averaging using the Foxtrot software (David and Pérez, 2009).

The size exclusion SAXS (SEC-SAXS) 1D intensities data were processed using BioXTAS RAW software (Hopkins et al., 2017) to extract the scattering profiles I(q) of each sample. All subsequent data analysis and modeling steps were carried out with BioXTAS RAW software, PRIMUS and other programs of the ATSAS suite (EMBL Hamburg, Germany) (Franke et al., 2017).

The SASREF (Petoukhov et al., 2012) program was used to perform quaternary structure modeling of the tetrameric HbF complex. For the SASREF rigid-body docking, P2 symmetry was used. The liganded structures of the α- and γ-subunits were obtained from the PDB entry 4MQJ (Soman and Olson, 2013). We imposed some contact conditions based on known contacts in HbF to help the convergence of the docking simulation. The contact conditions are summarized in the Supplementary Material.

### Circular Dichroism

Circular dichroism (CD) measurements were performed at 20°C in a Jasco J-815 Circular Dichroism spectropolarimeter. The samples were prepared in freshly degassed 10 mM phosphate buffer pH 6.0 and loaded into a 0.1 mm path length cuvette. The data was collected between 300 and 179 nm, and three scans were averaged for each sample.

### Animal Studies

The wtHbF and double mutant αA12D/A19D were selected for the animal study. The samples were subjected to endotoxin removal measures before the experiment, passing each sample through two high capacity Proteus NoEndo™ spin columns (Vivaproducts) according to the manufacturer's instructions. The protocols used were approved by the Institutional Animal Care and Use Committee at Malmö/Lund, Sweden. The animal study was performed by supplying a top addition of Hb. Female Balbc mice (Janvier, Le Genest-Saint-Isle, France) were injected with 5 mg of Hb (volume ≤ 0.2 ml), with five replicates (*n* = 5) per time point. Physical data such as weight and body temperature were recorded, and samples were taken at four different time points after intravenous injection (5 min, 2, 6, and 24 h). With four time-point groups per Hb variant plus non-injected control group (*n* = 5) for both wtHbF and αA12D/A19D series, a total of 50 mice were used in this study. The samples were frozen and stored at −80°C before pharmacokinetic evaluation. Enzyme-linked immunosorbent assay (ELISA) was used to quantify wtHbF and αA12D/A19D in plasma and urine samples. Rabbit anti-HbF affinity-purified polyclonal antibody (“Bonita,” Agrisera AB, Sweden) was used to capture the recombinant HbF proteins onto microtiter plates (NUNC, MaxiSorp, VWR). After thorough washing, horseradish peroxidase-conjugated sheep anti-HbF polyclonal antibody (Bethyl Labs, TX, USA) was incubated for detection of the captured HbF. Tetramethylbenzidine (TMB single solution, Invitrogen) was used to develop the plates, and the absorbance was measured at 650 nm in a SpectraMax® M2 Microplate Reader (Molecular Devices LLC). Standard curves of the recombinant proteins were prepared alongside the samples in all plates. Least-square fitting to an exponential decay equation with the Solver function in Excel (Microsoft) was used to calculate the recombinant Hbs half-life in plasma. Mouse albumin levels in urine were quantitated using a commercial kit (Mouse Albumin SimpleStep ELISA Kit, Abcam, UK), according to the manufacturer's instruction.

### Data and Statistical Analysis

The experimental data were collected in at least three repeats (*n* = 3) for each sample, unless otherwise stated. The Microsoft Excel software with the Solver add-in was used for least square fittings of experimental reactions to the time courses. The statistical analysis was done in the Jamovi software (Jamovi, 2020) with independent samples *t*-tests, and values are reported as mean ± SD. A *p* < 0.05 was considered statistically significant to report as possible differences.

## Results

### Basic Functionality of HbF Mutants

The three mutants of HbF were obtained by site-directed mutagenesis and confirmed by Sanger sequencing (GATC, Germany). Expression levels of all mutants were similar to wtHbF, yielding around 10–15 mg purified Hb/L culture, and comparable to previously reported yields of recombinant HbF (Ratanasopa et al., 2016; Kettisen et al., 2018).

**Figure 2:**
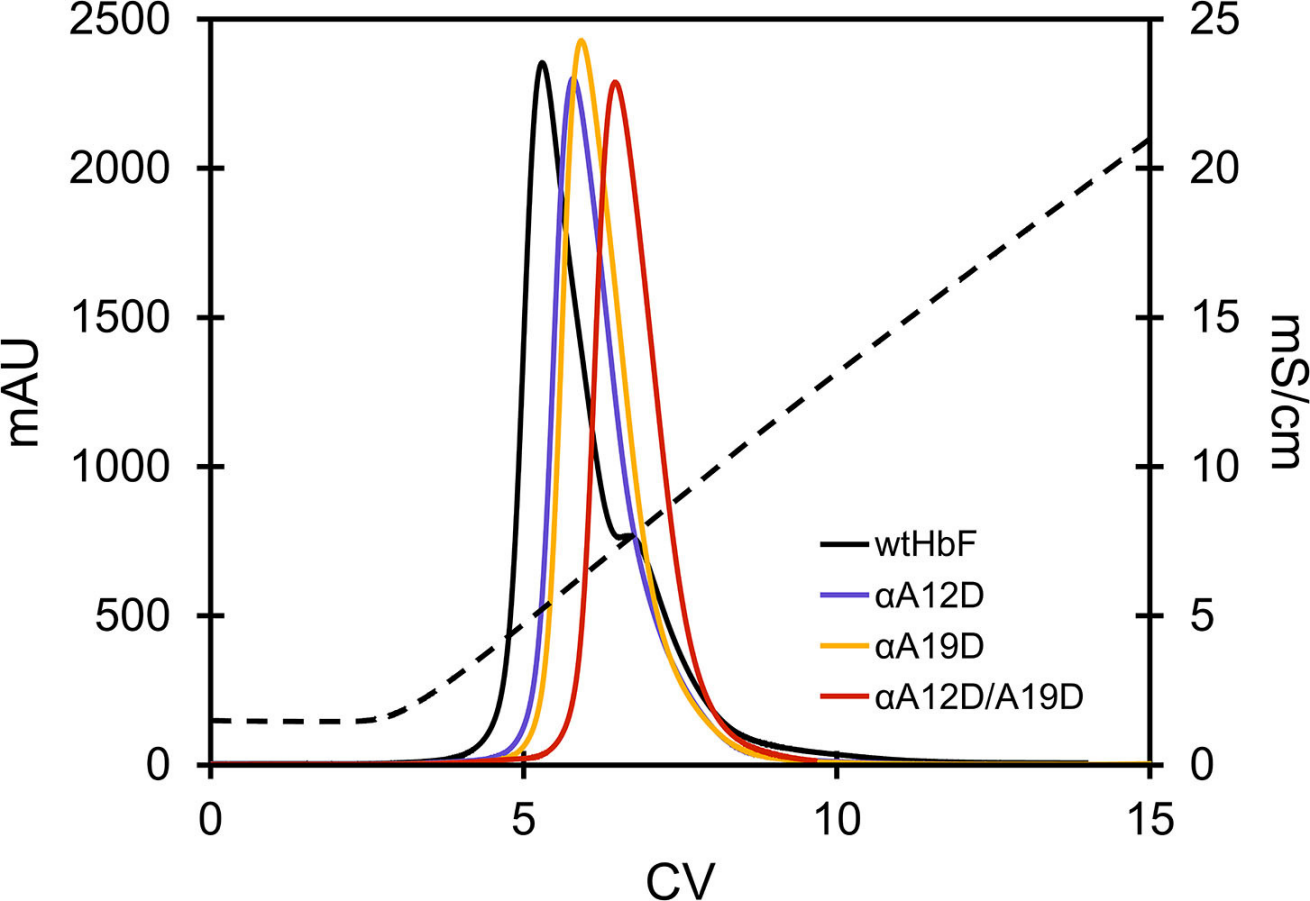
Chromatogram of the elution step of wtHbF and mutants αA12D, αA19D, and αA12D/A19D on a HiTrap Q HP column. The column was equilibrated with 20 mM Tris-HCl pH 7.4 and the dashed line shows the change in conductivity (mS/cm) as the NaCl concentration was increased over a linear gradient.

All proteins were isolated from bacterial extracts using the same protocol. During chromatographic purification, especially the double mutant notably displayed some visual spreading during the binding step to the cation exchange resin compared to wtHbF. The dynamic binding capacity (DBC) of the double mutant was thus significantly lower than wtHbF and was only 4.4 mg/ml at 10% breakthrough. Another difference was observed during the gradient elution in the anion exchange chromatography step, as the proteins differed in their elution retentions, with the mutant proteins having elution peaks at higher conductivity than wtHbF (Figure 2, Table 1).

**Table 1:** Anion chromatography elution data, pI determination (*n* ≥ 2), autoxidation rates, and heme loss rates of wtHbF, αA12D, αA19D, and αA12D/A19D (IEF gel picture can be found in the Supplementary Figure 2).

	**wtHbF**	**αA12D**	**αA19D**	**αA12D/A19D**
Cond. at peak max (mS/cm)	6.1 ± 0.2	6.8 ± 0.1	7.1 ± 0.2	7.7 ± 0.1
Isoelectric point	7.1	6.7	6.8	6.6
Autoxidation rate (h^−1^)	0.0035 ± 0.0005	0.0033 ± 0.0002	0.0033 ± 0.0002	0.0034 ± 0.0002
Heme loss rate (h^−1^)	0.99 ± 0.11	1.12 ± 0.15	1.01 ± 0.15	1.13 ± 0.09

The spectral examinations of the HbF mutants revealed that all different forms showed the same absorbance peaks as expected from functional human Hb (Supplementary Figure 1). As expected, the pI determinations showed significant differences between the mutants, and the value decreased with added aspartate residues (Supplementary Figure 2). The results from the autoxidation experiment also indicated no differences between the wtHbF and the mutants, as the rates were found to be similar. Also, the heme loss rates showed no significant differences. Table 1 summarizes the results. In general, the results indicate that the mutants retained basic functionality of the wtHbF, confirming that none of the mutants appeared to have altered ligand binding performance or caused evident structural instability affecting the heme.

### DNA Cleavage

**Figure 3:**
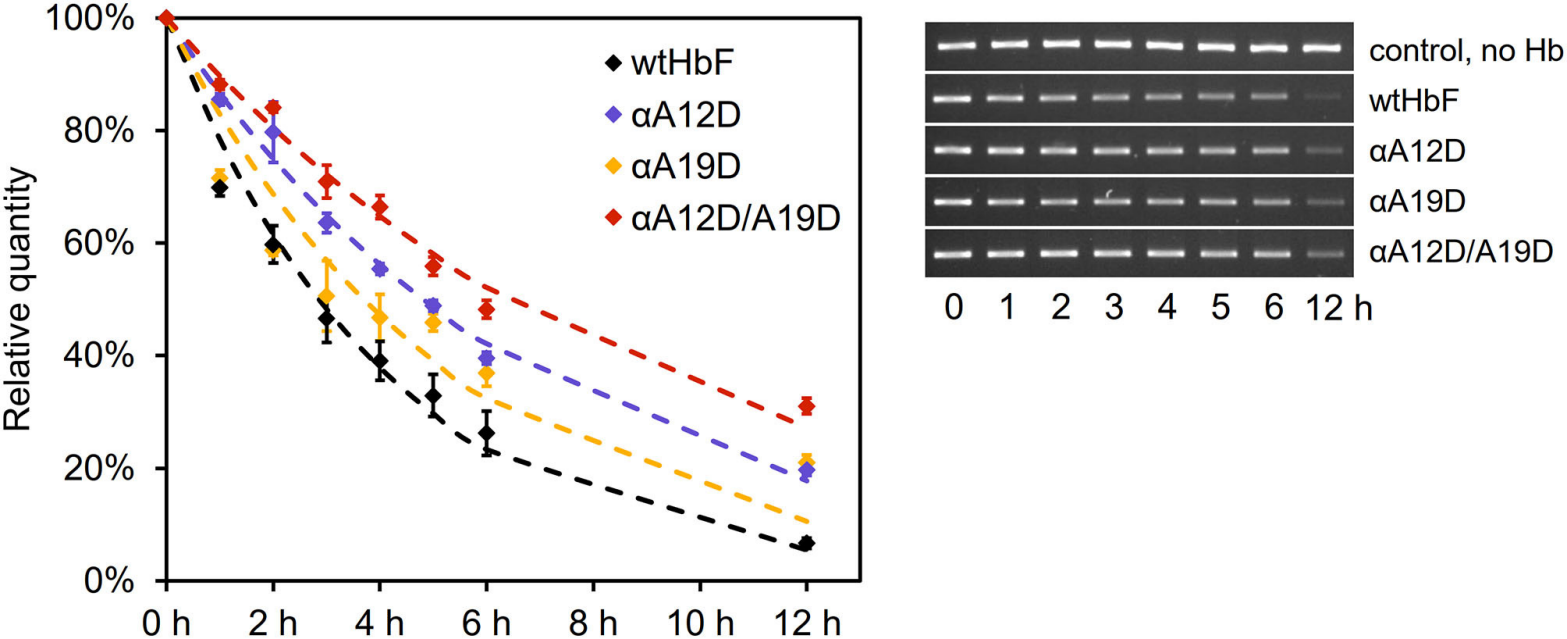
DNA cleavage of 3 ng/μl pUC18 plasmids incubated with 75 μM Hb of the different HbF mutants at 37°C in 20 mM phosphate buffer pH 7.2. The graph shows the mean values of the remaining DNA content, with error bars representing the standard deviation, together with the fitted curves of the exponential decay (dashed lines). Statistical differences were determined by independent *t*-test (*p* < 0.05). The pictures of the gel bands show the supercoiled band over time.

Cell-free Hb may have detrimental effects on many biomolecules. Proteins, lipids, and nucleic acids are often attacked by hydrolytic and oxidative side-reactions (Simunkova et al., 2019). In this study, we examined the hydrolytic effects of HbF on isolated plasmids. This reaction can monitored by following the conversion of supercoiled DNA to the open circular conformation. The result from the DNA cleavage assay is shown in Figure 3. Without any Hb added, no deterioration/cleavage of the supercoiled plasmid band was detected throughout the experiment. However, in the presence of Hb, the band was readily cleaved over time. Reduced cleavage rates for all three mutants were seen compared to wtHbF, with double mutant αA12D/A19D having the most supercoiled DNA remaining compared to the wtHbF at all time points. At the final 12 h sample, αA12D/A19D had 31% of the original signal left, while wtHbF only had 7%. The fitted decay constant for wtHbF was 0.24 h^−1^, compared to 0.19 h^−1^ for αA19D, 0.14 h^−1^ for αA12D, and 0.11 h^−1^ for αA12D/A19D. The double mutant's DNA cleavage rate was the slowest, with an observed 55% reduction of the cleavage rate compared to wtHbF.

### Thermostability

**Figure 4:**
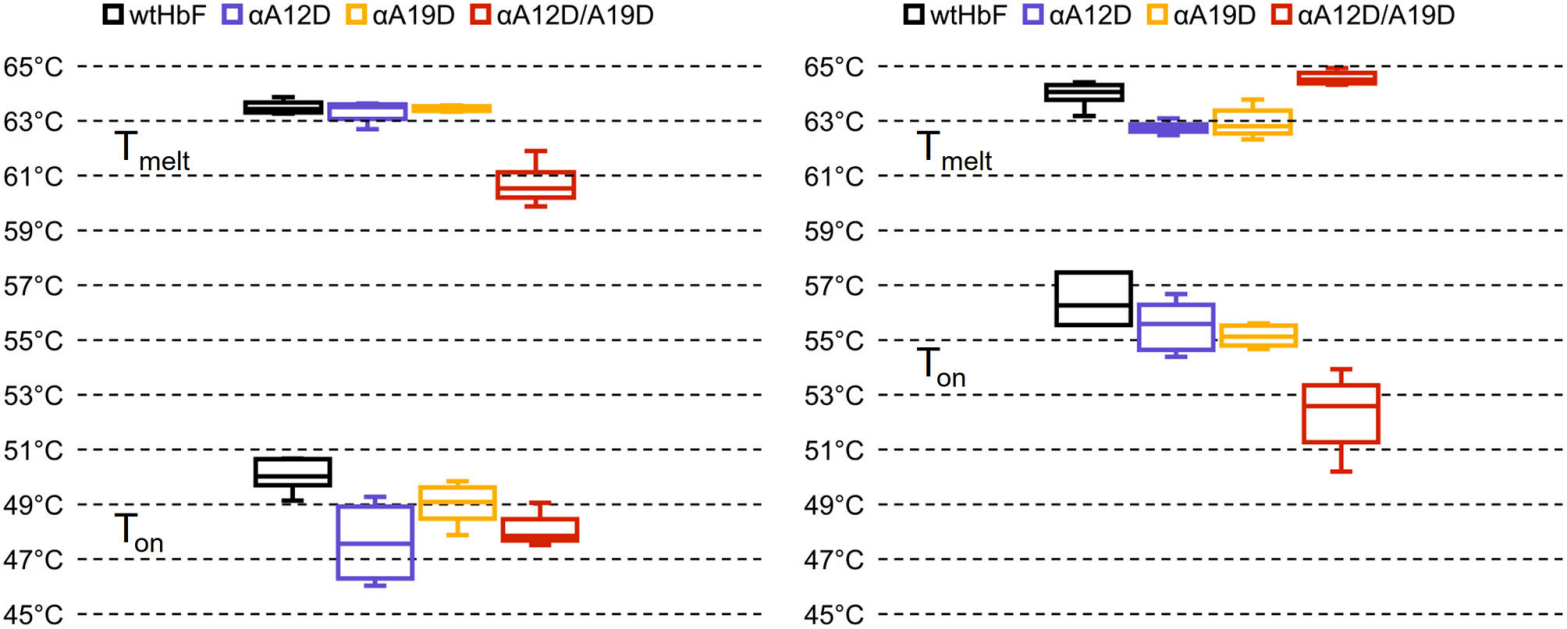
Box plots of the onset and transition temperatures of ferric **(Left)** and ferrous **(Right)** samples of wtHbF, αA12D, αA19D, and αA12D/A19D, calculated from intrinsic fluorescence at 350:330 nm ratio measured in a Prometheus NT.48 instrument (Nano Temper Technologies) over a temperature gradient. T_on_ denotes the onset of unfolding while T_melt_ signify transition temperature (50% unfolded). See Supplementary Figure 3 for the thermograms, and Supplementary Table 1 for independent *t*-test comparisons (*p* < 0.05).

The results of the thermal stability analysis in the Prometheus NT.48 instrument is presented in Figure 4 (in Supplementary Figure 3 shows the obtained thermograms and Supplementary Table 1 shows the *t*-test comparisons). Onset temperature (T_on_, start of unfolding) and transition temperature (T_melt_, 50% unfolded) were calculated by the PR.therm Control v.2.04 program. The largest difference could be seen in the T_on_ temperatures between ferric and ferrous O_2_-bound samples, with onset temperatures around 4–8°C higher for the ferrous samples. For the ferric T_on_ values, wtHbF had the highest temperature at 50.1 ± 0.6°C, single mutant αA19D had a higher T_on_ value than double mutant αA12D/A19D, but single mutant αA12D was not statistically different from either of the two other mutants. The T_melt_ for the ferric samples showed similar values for the wtHbF and the single mutants, around 63°C, while the double mutant displayed a lower value at 60.6 ± 0.7°C. For the T_on_ of the ferrous samples, wtHbF and the single mutants had similar values around 55–56°C, and the double mutant had a lower value observed at 52.3 ± 1.3°C. The ferrous T_melt_, on the other hand, showed that the double mutant had the highest T_melt_ at 64.4 ± 0.2°C, followed by wtHbF at 63.9 ± 0.4°C, while the single mutants had comparable values about one degree lower than wtHbF. In summary, unfolding starts at a lower temperature for ferric samples compared to ferrous O_2_-bound samples for all HbF variants, indicating increased susceptibility to structural change in the ferric state. T_melt_ is higher for ferrous samples of wtHbF and double mutant αA12D/A19D compared to ferric samples, and the opposite is seen for the single mutants where the T_melt_ is slightly lower for the O_2_-bound samples. In general, all mutants display minor differences compared to wtHbF, indicating only minimal structural perturbation imposed by the mutations.

### Small-Angle X-ray Scattering

Small-angle X-ray scattering was used to obtain structural information on the HbF tetramers. Guinier analysis and pair distribution function yielded the radii of gyration (R_g_) and maximum diameter (D_max_). The results for all the samples are summarized in Table 2. As expected, we found that the R_g_ and D_max_ were comparable, as supported by a Bonferroni comparison test suggesting no significant differences. The SEC-SAXS profiles and I(q) selection can be viewed in Supplementary Figure 4, while Supplementary Figure 5 display the scattering profiles for each sample.

**Table 2:** Scattering results.

**Sample**	**I(0)^a^**	**Rg (Å)^b^**	**Dmax (Å)^c^**
wtHbF	1.73 ± 8.82 10^−3^	24.21 ± 0.09	64
αA12D	2.39 ± 3.33 10^−3^	24.62 ± 0.05	71
αA19D	2.29 ± 7.42 10^−3^	24.67 ± 0.13	68
αA12D/A19D	3.12 ± 6.85 10^−3^	24.16 ± 0.09	67

a *Scattering intensity at zero angle*.

b *Radius of gyration in Å*.

c *Maximum size of the macromolecule Dmax in Å. See details in the Supplementary Material*.

A further comparison was performed using the whole scattering curves. The method uses a goodness-of-fit test, correlation map, for assessing differences between one-dimensional spectra independently of explicit error estimates, using only data point correlations (Franke et al., 2015). Table 3 summarizes the pairwise comparisons. It was found that the wtHbF was not different from the double mutant αA12D/A19D, but different from the single mutants. Moreover, the single mutants αA12D and αA19D were not significantly different from one another, but significantly different from the double mutant. A cursory inspection of the correlation maps suggested that the differences were located in the intermediate to higher q range. To further understand the effects of the mutations, we modeled the scattering curves using a rigid-body docking approach with the SASREF program.

**Table 3:** Correlation map—*p*-value at the 0.01 significance level.

**Sample**	**wtHbF**	**αA12D**	**αA19D**	**αA12D/A19D**
wtHbF		** <0.001**	**0.00739**	0.3842
αA12D			0.983	** <0.001**
αA19D				** <0.001**
αA12D/A19D				

*Correlation map analysis using Primus, ATSAS (Franke et al., 2015). Bold values indicate significant difference*.

#### Rigid-Body Docking

**Figure 5:**
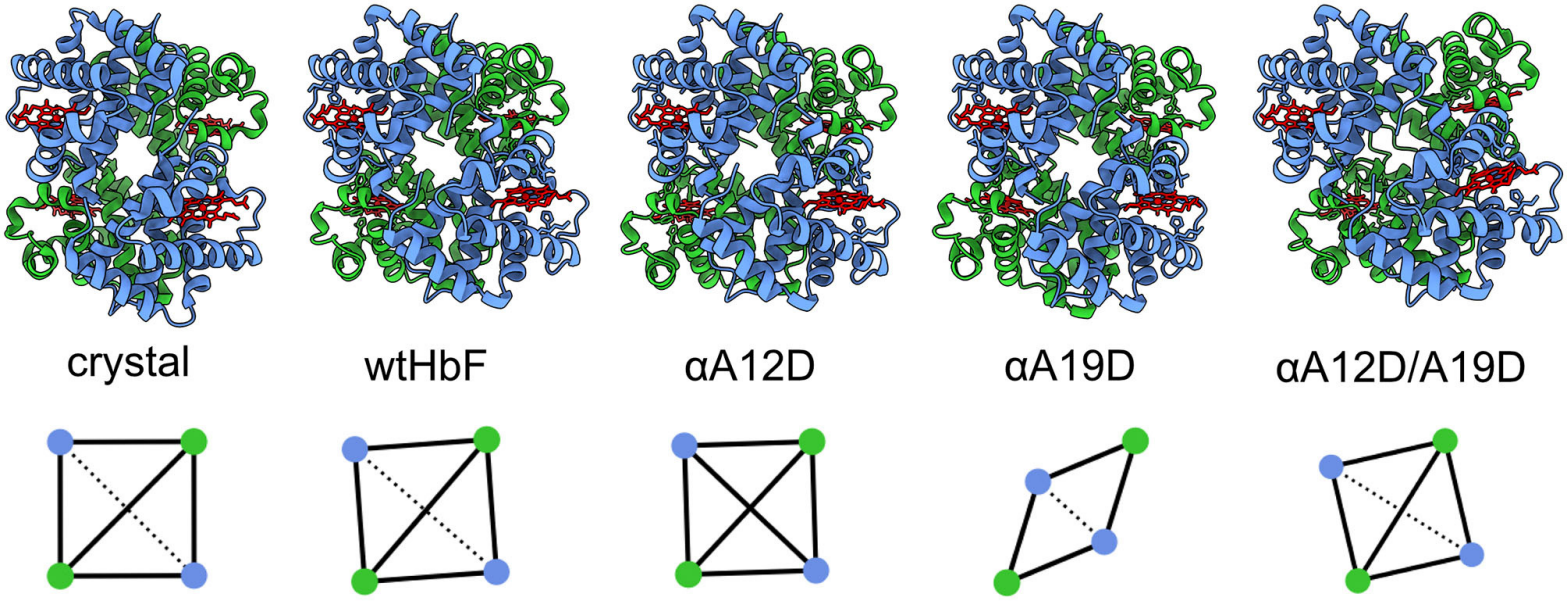
The crystal structure of pdb ID 4MQJ (Soman and Olson, 2013) and four 3D models of the recombinant HbFs generated by the rigid-body docking. The α-subunits are displayed in blue and the γ-subunits in green, while the heme groups are red. Below, the chain-chain diagrams calculated by ChimeraX software are shown for the different structures generated by the rigid-body docking (SASREF, contact condition 7 Å). From left to right: crystal structure wtHbF, in solution SAXS of wtHbF, αA12D, αA19D, and αA12D/A19D. Blue dots are the α-chains, and green dots are the γ-chains. The solid lines show major interface areas ≥300 Å^2^, and the dotted lines show minor interface areas.

Figure 5 display the 3D models obtained from the rigid-body docking with a 7 Å maximum contact condition. The fit to the scattering curves is found in Supplementary Figure 7. To quantify the degree of deformation of the tetramer and subunits, we used a chain-chain network diagram analysis. The diagram was generated based on the subunits in HbF (4MQJ) computed in the ChimeraX software (Goddard et al., 2018) using the interface command (UCSF, 2020). The chain–chain network diagrams are also displayed in Figure 5. A square-shaped chain-chain contact diagram is interpreted as a structure with the expected hemoglobin interfaces, as guided by the crystal structure. To assess the credibility of the models, the contact residues were compared with the contact residues found in the crystal structure. As seen in Figure 5, the chain-chain diagram was consistent between the crystal structure and solution scattering of the wild type; as well as the double mutant (αA12D/A19D). To test the reliability of the rigid-body docking results, the contact condition was increased from 7 to 9 Å. We did not observe a significant change in the scattering fits with the 9 Å condition (Supplementary Figure 8 for fits and Supplementary Figure 9 for 3D models and diagrams), however, the chain-chain contact diagrams worsened and produced unlikely contact interfaces, especially for wtHbF and αA19D, indicating that the 3D structure analysis with the chain-chain contacts is imperative to evaluate the rigid-body models. This further suggests that the scattering curve fittings can fit well even to improbable structure conformations, and that the structural change caused by the mutations were too small to be decisively determined within the resolution of the SAXS data.

### Circular Dichroism

**Figure 6:**
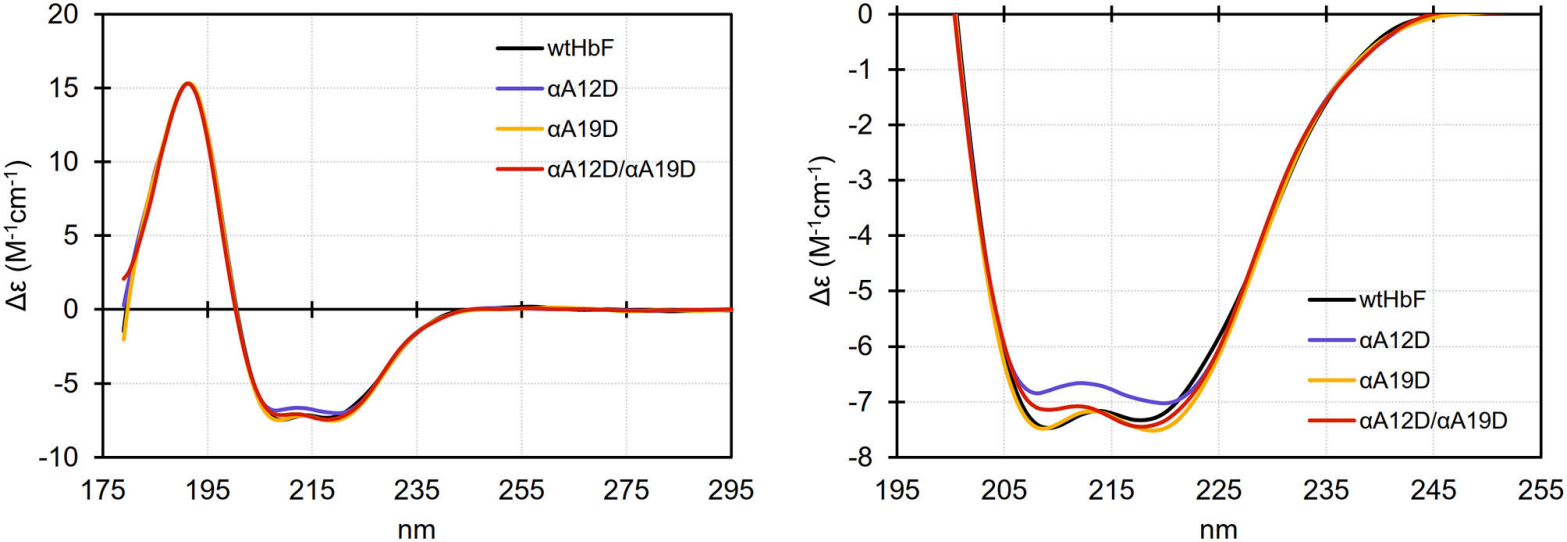
Circular dichroism spectra (scaled at 191 nm and smoothed) of wtHbF, αA12D, αA19D, and αA12D/A19D, respectively. The samples were prepared in 10 mM phosphate buffer pH 6.0 and the measurements were done in a Jasco J-815 Circular Dichroism spectropolarimeter at 20°C using a 0.1 mm path length cuvette. Left panel shows the full spectral range, and the right panel shows a magnified view of the spectral range covering the characteristic dual valley shape of alpha-helical structures.

The scattering analysis in the previous section suggested that the overall parameters (i.e., size and shape) are similar for the wtHbF and the mutants, but a detailed analysis of the scattering curve suggested deformations of the 3D structure to improve the fits to the scattering curve in the middle to high q range. A simple interpretation for the needed deformation would be some changes in secondary and possibly tertiary structures. We, therefore, measured the wtHbF and mutants using circular dichroism. Figure 6 summarizes the findings. The data were scaled and smoothed for better comparison.

The dominant alpha-helical structures of Hb were clearly visible in the CD measurements. The magnified circular dichroism comparison suggested that all of the mutants showed small differences compared to the wtHbF, i.e., the spectra did not entirely overlap. Specifically, the magnitude of Δε at both 209 and 218 nm was decreased for the αA12D mutant, while the αA19D mutation appeared to cause a widening and deepening of the 218 nm valley. The double mutant appeared to display features from both mutations, showing a decreased magnitude of Δε at 209 nm, and an increase at 218 nm, compared to wtHbF. Online secondary structure prediction tools predicted that the wtHbF had a little bit higher fraction of alpha-helical structures compared to the mutants (Louis-Jeune et al., 2012; Wiedemann et al., 2013; Micsonai et al., 2015), possibly suggesting some degree of deformation affecting the ratio of helix structures. The complexity of the underlying structure precluded any further analysis. We concluded, however, that the deformation needed to fit the scattering curves could be related to secondary structure change or reorganization.

### Animal Studies

**Figure 7:**
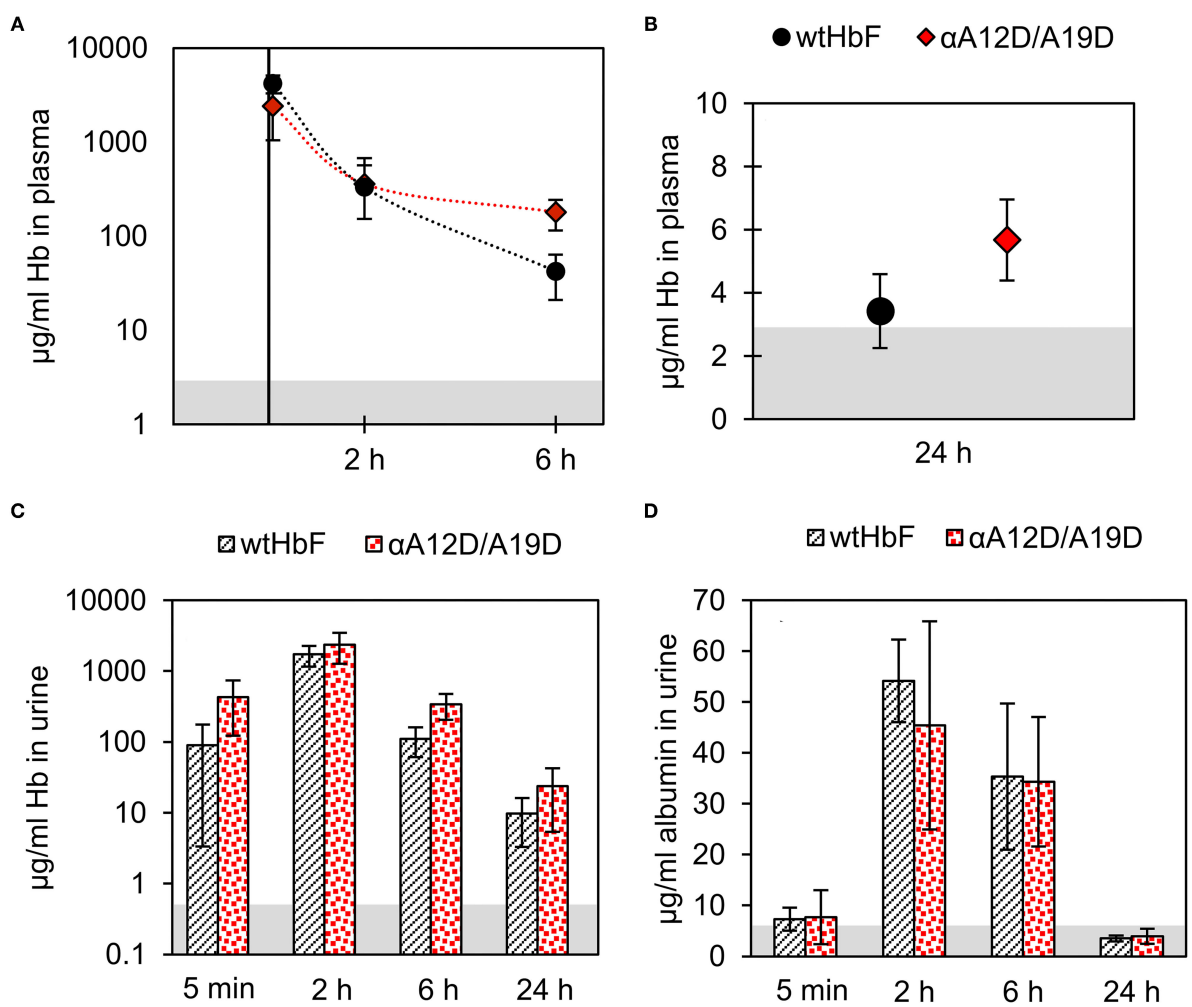
The pharmacokinetics of wtHbF and the αA12D/A19D mutant were analyzed in plasma and urine samples at four different time-points (5 min, 2, 6, and 24 h, *n* = 5 per time point and Hb variant) by ELISA. Data is presented as mean ± SD. Differences are determined by independent *t*-tests with *p* < 0.05 significance level. The gray area in the graphs shows the signal of the non-injected control (*n* = 5). Panels **(A)** and **(B)** show the results of the plasma analysis, and no difference is seen for the two first time points at 5 min and 2 h, but at 6 and 24 h, significantly more αA12D/A19D mutant is detected compared to wtHbF. **(C)** In urine, the concentration of Hb was found to be similar between the mice injected with either wtHbF or αA12D/A19D, except for at 6 h, where a significant difference is seen with αA12D/A19D having a higher concentration than wtHbF. **(D)** The albumin assay showed that the concentration of albumin in the urine with either protein variant was similar at each time point.

All test animals displayed a healthy appearance after injection of either wtHbF or αA12D/A19D, and no significant differences in weight or body temperature were found compared to the untreated control. One sample out of the total 50 had to be discarded due to insufficient/malfunctioning injection, resulting in four replicates instead of five for the 5 min sample group for wtHbF. The analysis of the plasma samples by sandwich ELISA showed that no significant difference in Hb concentration could be detected between wtHbF and αA12D/A19D for the two first time points. However, at 6 and 24 h, the αA12D/A19D mutant had higher concentrations remaining in the plasma compared to the wtHbF, as seen in Figures 7A,B. Moreover, for the wtHbF 24 h time point, it was found that the wtHbF level could not be distinguished from the control, i.e., the protein did not remain in the plasma at detectable levels at 24 h. The calculated half-life of wtHbF and αA12D/A19D was 38 and 46 min, respectively. However, from the data points, it appears that the process possibly might be better represented by two phases, with a faster elimination rate in the first 2 h, followed by a slower phase after 2 h. To further characterize plasma clearances, Hb levels were also analyzed in urine samples. The Hb ELISA assay of the urine samples, Figure 7C, showed similar Hb concentration at all time points except for at 6 h, where the double mutant had a slightly higher concentration than wtHbF. This finding support the plasma data regarding the double mutant having higher concentrations remaining in circulation for longer than wtHbF. In the mouse albumin assay, it was found that the albumin levels in the urine were increased after Hb administration, and the concentrations were similar at all time points for wtHbF and the double mutant, as seen in Figure 7D. Increased albumin presence in urine indicate challenged renal functions, and the overall glomerular filtration thus seems to be equally affected by the wtHbF and the double mutant.

## Discussion

Our findings confirm the potential value of adding negative charges onto to the surface of recombinant HbF. The presence and visibility of the surface-exposed charges were established through liquid chromatography and IEF. The DNA cleavage rate was reduced with the added negative charges, and the double mutant αA12D/A19D stayed in circulation longer than wtHbF in a top addition of Hb in a mouse model. At the same time, spectral examination, autoxidation, and heme loss studies showed that the mutations did not adversely disturb the basic structural and functional properties of HbF.

To put this work in perspective, rational mutagenesis has been used to investigate various aspects and property altering strategies on Hb in the field of artificial oxygen carriers (Varnado et al., 2013), mainly toward the goal of realizing a globally accepted oxygen therapeutic based on this protein. When HBOCs are realized, they are normally either chemically cross-linked or modified by polyethylene groups to increase the molecular weight of Hb. This increases the physiological circulation times, but the modifications often require substantial additional work efforts. In addition, these processes often generate heterogeneous products with mixed physiological behavior. Research on HBOCs has to date mainly focused on several properties of the Hb moiety, such as structural stability, oxidative reactivity, and nitrogen oxide scavenging, and several review papers have summarized the development and toxic issues of HBOCs (Alayash, 2017; Estep, 2019; Ferenz and Steinbicker, 2019). In this study, we have addressed an alternative strategy based on introducing negative charges on the surface of Hb. Such modifications reduce cellular uptake due to repulsion with the negatively charged surface of cells and tissues. As an evolutionary designed intracellular protein that prefers tight packing at high concentrations, the pI of wild-type Hb favors minimized surface charge repulsion at physiological pH. However, for this protein to be applied extracellularly, modification to fit the plasma environment is crucial. Applying protein engineering to adjust and optimize net charge to this end could be a worthwhile strategy to consider alongside other property-enhancing engineering strategies on Hb for HBOC development. Furthermore, the introduction of negative charges on Hb would reduce interactions with essential biomolecules such as nucleic acids.

The selection of mutation sites for introducing negative charge on the HbF surface was primarily focused on the A helix since the surface exposed residues in this part of the protein are located far away both from the heme pockets and from the contact surfaces between the subunits of the tetrameric Hb structure, according to existing crystal structures of wild-type HbF (Frier and Perutz, 1977; Soman and Olson, 2013). We selected the two sites included in this study due to reports of natural mutations from alanine to aspartate at these sites have appeared to be stable and not associated with clinical symptoms (Rosa et al., 1966; Trincao et al., 1968; Rahbar et al., 1976; Dash and Huisman, 1988). Assuming minimal structural distortion, the difference between the sites is that αA12D is located within the A helix, while αA19D is located in corner between the A and B helix. Comparing them as single mutants and together in the double mutant gave the opportunity to examine the suitability of placement of negative charges. These modifications are also distantly removed from both the heme binding pocket and the dimer-dimer interaction surface. This latter area is the binding location for the serum protein haptoglobin. When examined *in vitro*, hemoglobin-haptoglobincomplex formation was not affected by the mutation sites used (Alayash et al., 2013; Ratanasopa et al., 2013).

In this study we included only modest increase in net charge by one and two additional charges per α-subunit, but supercharging proteins toward extensive positive or negative surface net charge have previously demonstrated promising plasticity of protein surfaces for this type of modification (Lawrence et al., 2007; Ma et al., 2020). As cellular membranes generally have a negative net charge, a positive surface net charge promotes association to cell surfaces and cellular uptake. Supercharged proteins with a high positive net charge exemplifies this (Cronican et al., 2011). We have focused on adding negative charge, but the strategy of increasing net charge toward a more negative surface to decrease nucleic acid interactions and cell surface association may, however, inadvertently promote interactions with positively charged biomolecules. Specific studies would be necessary to examine such effects, both within in the heterologous host cell during expression and in the intravascular space. In this study, with only minor increased negative net charge being examined, we have not detected any apparent change in interaction with the host cell proteins, i.e., the HbF mutant variants were purified from the *E. coli* extract without persistence of any unexpected contaminant. Studies on charged nanoparticles and the formation of protein corona surrounding these particles in the intravascular space may indicate possible interacting partners with hypothetical supercharged Hb variants (Sakulkhu et al., 2014).

*Chromatography is sensitive to surface-exposed modifications of HbF*. The results of this study clearly show that the mutations contributed to a more negatively charged HbF molecule. The observations in the liquid chromatography purification point to altered surface interaction due to the additional negative charges, exhibiting increased repelling from the negatively charged resin and increased interaction with the positively charged resin. Although readily noticeable, the elution peaks in the anion resin displayed relatively small shifts toward higher salt concentrations and would probably not suffice to completely separate the mutants from wtHbF if mixed in the same sample on this resin. In another study, we used an alternative kind of resin (molecularly Hb-imprinted polymer beads) which is much more specific than commercial ion exchange media (Zhang et al., 2019). Significant differences between wtHbF and the three mutants were seen as the mutant elution peaks were shifted to a considerable extent, and more clearly separated the mutants from wild type. Different types of chromatography resins thus present quick ways to screen the impact of surface-located strategies on the HbF protein.

*Increased negative net charge reduce adverse interactions with DNA*. The DNA cleavage assay further solidified the conclusion that the mutations chosen for this study significantly contributed to a more negative surface of the HbF. In this study, we used supercoiled plasmids as models for examining hydrolysis of DNA. The reason behind this choice of nucleic acid substrate is based on the facile detection of single-stranded nicks. When such nicks are formed, the supercoiled molecules are converted to an open circular conformation that readily can be detected on agarose gels. This provides a simple tool for detecting breaks in a single DNA strand before the DNA molecule is fully cut. Hbs are alpha helical proteins and may thereby bind to the major groove of DNA. When negative charges are introduced on the surface of HbF, the interaction between the protein and the DNA is partly obstructed. The cleavage of the pUC18 plasmid was thus decreased with just a single mutation, and even more with two negative mutations. Assuming that increased negative net charge of the protein repels the negatively charged phosphate backbone of DNA, this is an indication that the strategy can decrease harmful interactions with important biomolecules or structures with a negative net charge. It can also be hypothesized that the mutants this way are less harmful to the host as they accumulate in the *E. coli* during recombinant expression, where the recombinant DNA is present together with the expressed Hb. From a cultivation perspective, this could be beneficial for yield during production as well. In fact, in a forthcoming study we modified the cultivation protocol to increase yield for recombinant HbF in shake flasks, and we found a better yield with the double mutant compared to the wtHbF. The reason for not detecting the difference with the HbF production protocol used in this study could possibly have been due to cultivation parameters limiting the expression in *E. coli*. Other hypothesized positive effects of adding negative charges, such as; increased solubility of the α-subunit and/or increased association to the γ-subunit, alongside the decreased toxic effect by reduced DNA cleavage activity seen in this study; could have been overshadowed by limiting cultivation parameters as determining factors for the yields in this study. Besides interactions between DNA and HbF, other effects *in vivo* can be expected in terms of modified protein-protein interactions, metabolite-protein binding etc. The metabolic consequences of these remain to be evaluated.

*Oxidative stability retained but thermal stability reveals possible minor destabilizing effect imposed by the mutations*. The retained oxidative stability and unaffected heme loss rates in combination with the positive effects of the added negative charges show valuable potential of the mutation strategy. Modification of Hb for HBOC applications often leads to increased oxidative reactivity (Meng et al., 2018), which ultimately contributes to the toxic side effects associated with these products. The mutation sites in this study are far from the heme pocket, so any influence on the oxidative stability or heme stability in this study would be due to structural perturbation affecting other sites in the protein, but no such effect was supported by our results. The thermostability study suggested on the other hand that the mutations may have an impact on structure that is visible during a temperature gradient. Notably, the double mutant αA12D/A19D in the ferric state appeared to show an increased instability. Heme loss studies are usually performed with ferric samples due to the increased heme release induced by the ferric state (Kassa et al., 2016). In line with our findings for the ferric samples in the thermal stability, the double mutant did show the highest mean heme loss rate of the HbF variants, but the measured rate was not found to be significantly different from wtHbF. Speculatively, one might hypothesize that there could be more risk of structural distortion from having both aspartate mutations together in the α-subunit, as they are relatively close to each other. More in-depth structural information is required to clarify the detailed structural impact, for example by NMR studies or X-ray crystallography, but the aspartate mutations seem to have imposed some minor changes which at least were noticeable in thermal denaturation studies. Nonetheless, the oxidative stability is not affected by these modifications.

*Structural studies support only minor local structural changes induced by the mutations*. The in-solution SAXS studies confirmed the integrity of the overall tetramer structure associated with HbF for all proteins. None of the mutants in this study had visible differences in terms of shape or dimension. The intermediate to high q range, albeit noisy, suggested small differences which we tried to model in a rigid-body docking with subunits of HbF obtained from a crystal structure (Soman and Olson, 2013). We had to employ contact conditions to align subunits, but even so, the insufficient resolution of SAXS could not decisively support the modeled structures to clarify the understanding of the structural effects induced by the mutations. Circular dichroism provided a little more insight as we could ascertain that changes to the secondary structure may play a role in the indistinct differences seen in the SAXS scattering data. Considering the substitution from wt alanine, a residue which is deemed to have one of the highest helix forming propensities, to the mutant aspartate, which has a much lower tendency (Pace and Scholtz, 1998), it is perhaps not surprising to see that the secondary structure examination by CD for the mutants show small differences. A substitution from alanine to aspartate is not a guarantee for destabilizing an alpha helix in a protein structure, given that other residues in nearby structure can stabilize the change. Otherwise, aspartate would favor coil formation over helix. The effect may be the case especially for the double mutant and single mutant αA12D, which have the substitution located within the A helix (A10), regarding the examination of the CD data. As the thermal denaturation alluded to structural differences for the mutants as well, especially for the double mutant, one may speculate regarding the suitability of the placement of negative charges. Several mutations need to be present to achieve a considerable change in overall net charge, and so it might be worthwhile to consider having the mutation sites further apart. Alternatively, having other types of residue substitutions to retain helix stability while adding negative, surface-exposed charges to HbF. Glutamic acid is another option for providing negative charge at physiological pH, and this residue has a higher alpha helix propensity due to the increased side-chain length (one extra-CH_2_-group) compared to aspartic acid. Further studies into the strategy of adding negative charges to the HbF will explore these alternatives.

*Double negative mutations extended circulation of HbF in a mouse model*. The animal studies with the wtHbF and the double mutant αA12D/A19D showed increased plasma half-life with the mutant compared to the wild-type protein. This indicates that the negative exposed charges at the mutation sites did, to some extent, alter the interactions related to the clearance of Hb from circulation. Although, examination of the data may indicate two phases of removal, with a first phase is dominated by more rapid elimination, and the second phase exhibiting slower elimination. As the mutants in this study had no tetramer-stabilizing cross-linking modifications, a possible explanation to the two phases observed could be that the rapid initial phase was predominantly driven by dimer formation and the subsequent enhanced removal of the dimer species (Bunn et al., 1969). The half-life found for wtHbF in this study was comparable to previously reported values of recombinant HbA administrated in a mouse model (Bobofchak et al., 2003). It is worth to note that the half-life of αA12D/A19D in plasma found in this work, albeit increased, still was relatively short. Combined with the albumin analysis of the urine samples showing that glomerular filtration is affected to a significant extent despite the added negative charges, further efforts are needed to improve the performance of modified HbF. The albumin levels at the later time point 24 h however suggest that the increased albumin leakage into urine was transient. More added negative charges and/or in combination with other plasma retention time extending protein engineering strategies should be investigated.

## Conclusion

In conclusion, the aim behind the work in this study was to focus on the effects of directly modifying the surface charge of the HbF protein by substituting non-polar alanine residues in the α-subunit into aspartate residues carrying a negative charge. Our results suggested that substitution to negatively charged, surface-exposed aspartate residues on the α-subunit of HbF forms stable mutants with no apparent adverse effect on normal functionality. The mutants show desirable properties such as decreased DNA cleavage as well as increased circulatory half-life in a mouse model for the double mutant αA12D/A19D. In terms of stability, especially for the ferric state, it might be interesting in further studies to see if a mutant HbF protein with more than one or two extra negative charges can retain stability by having the mutation sites more spread out across the surface of the whole protein. Thus, other mutation sites and/or residues could be interesting to examine for this strategy. Overall, these results present a protein engineering strategy for Hb that displays promising properties that contribute to the goal of improving the global functions of HbF for adaptation into a functional oxygen therapeutic. It remains to be evaluated if larger effects can be obtained by introducing more charges on the protein surface.

## Data Availability Statement

The original contributions generated for the study are included in the article/Supplementary Material, further inquiries can be directed to the corresponding author/s.

## Ethics Statement

The animal study was reviewed and approved by Institutional Animal Care and Use Committee at Malmö/Lund, Sweden.

## Author Contributions

LB and KK initiated the project. KK designed and performed the experiments except for the SAXS measurement (CD) and the animal handling (ES). KK, CD, and ES analyzed the data. KK wrote the manuscript, with contribution from CD. All authors read, commented on, edited, and approved the final manuscript.

## Conflict of Interest

The authors declare that the research was conducted in the absence of any commercial or financial relationships that could be construed as a potential conflict of interest.
