# Supplementary material for: Site-Specific Introduction of Negative Charges on the Protein Surface for Improving Global Functions of Recombinant Fetal Hemoglobin
Source: Front Mol Biosci. 2021 Mar 30;8:649007. doi: 10.3389/fmolb.2021.649007 (PMC8042259; doi:10.3389/fmolb.2021.649007)
Supplement: Supplementary file 1 [file Table_1.DOCX]

Supplementary Material

Site-Specific Introduction of Negative Charges on the Protein Surface for Improving Global Functions of Recombinant Fetal Hemoglobin

# Supplementary Data


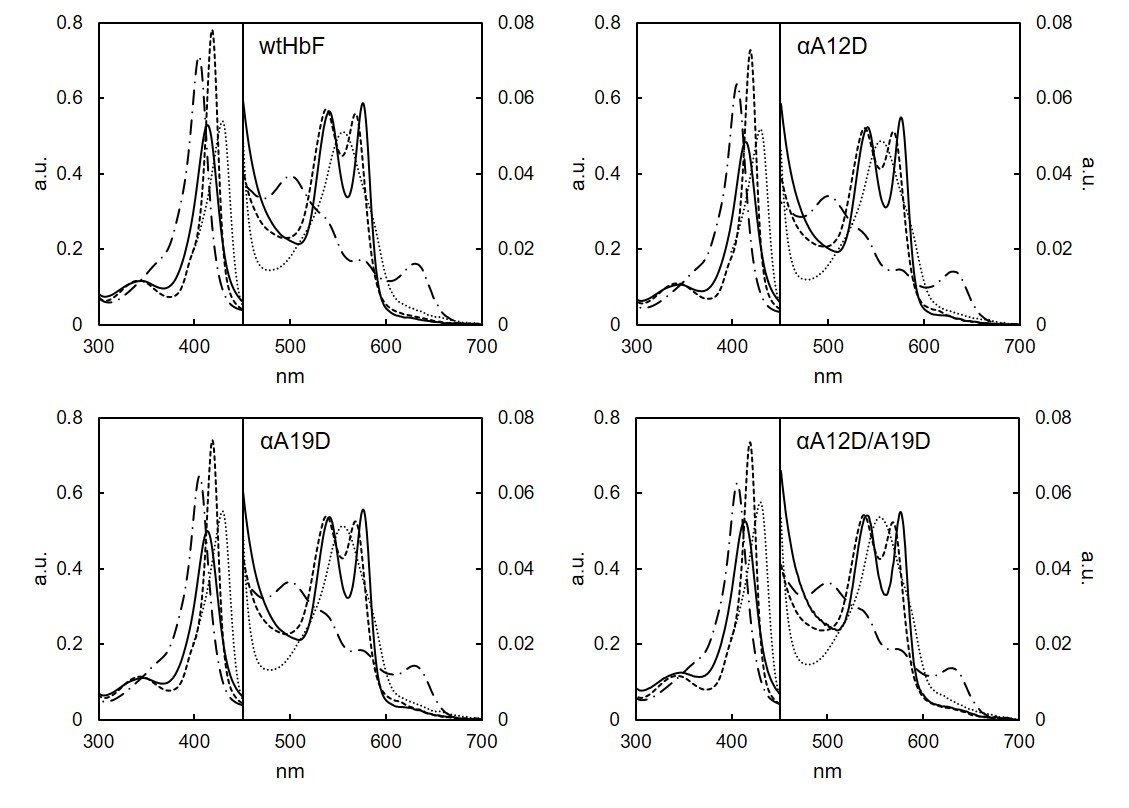


**Figure S1.** Spectra of recombinant fetal hemoglobin variants – wtHbF, αA12D, αA19D, and αA12D/A19D. The solid line represents Hb bound to oxygen, the dotted line represents deoxy Hb, the dashed line represents Hb bound to CO, and the dashed-and-dotted line represents ferric Hb (Fe^3+^).


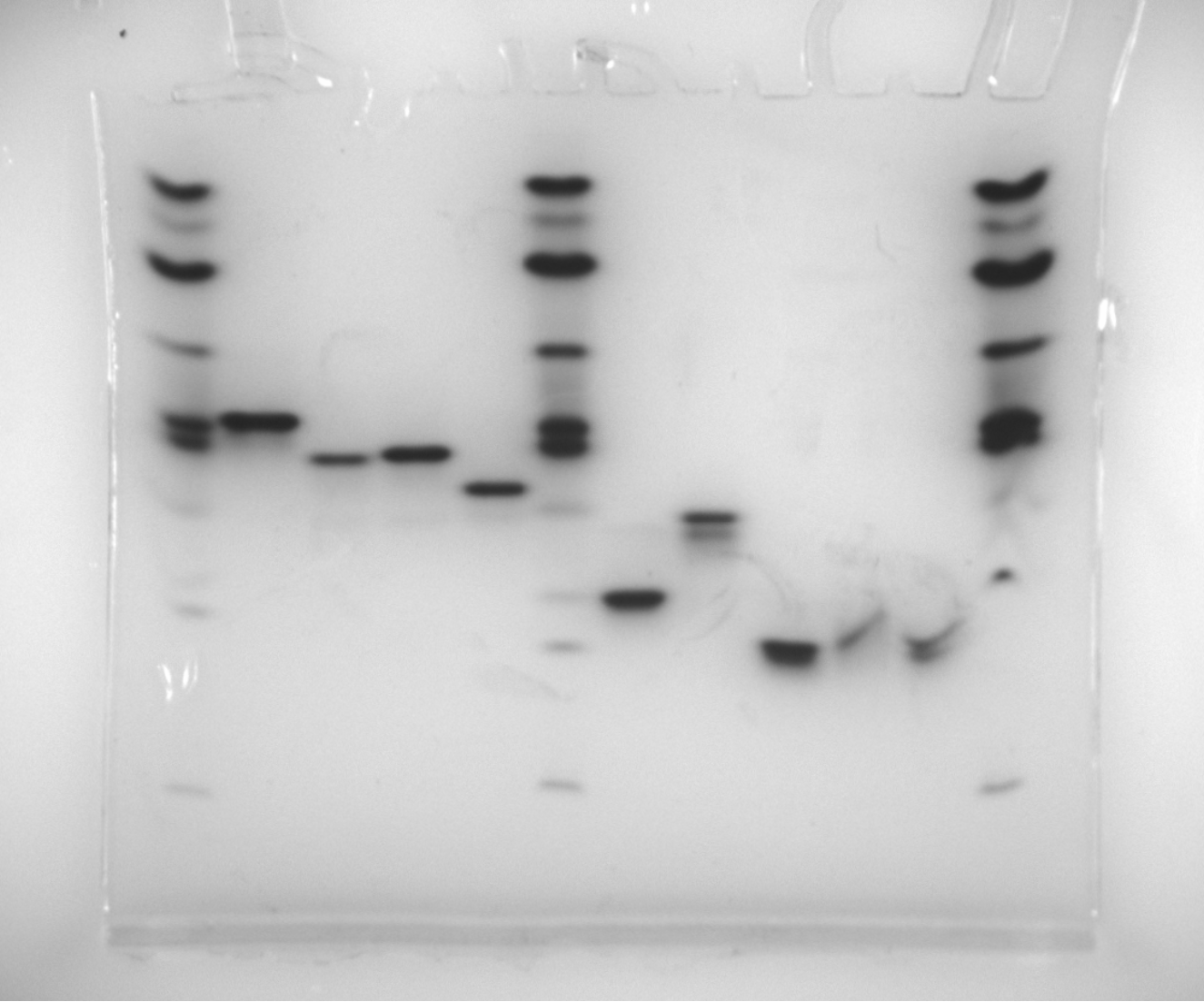

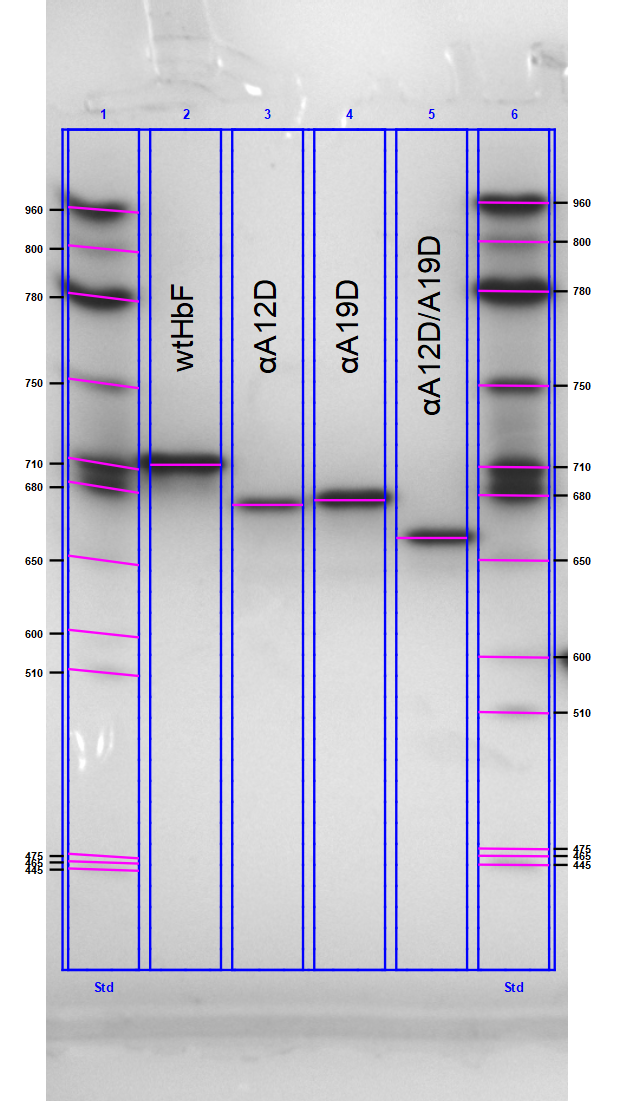


**Figure S2.** Isoelectric focusing of wtHbF, αA12D and αA19D, and αA12D/A19D on a Novex^®^ pH 3-10 IEF gel (Invitrogen). The pI is determined with Bio-Rad’s IEF Standards in well 1 and 6. Left: cropped gel with the relevant samples, with marked bands. Right: entire gel.


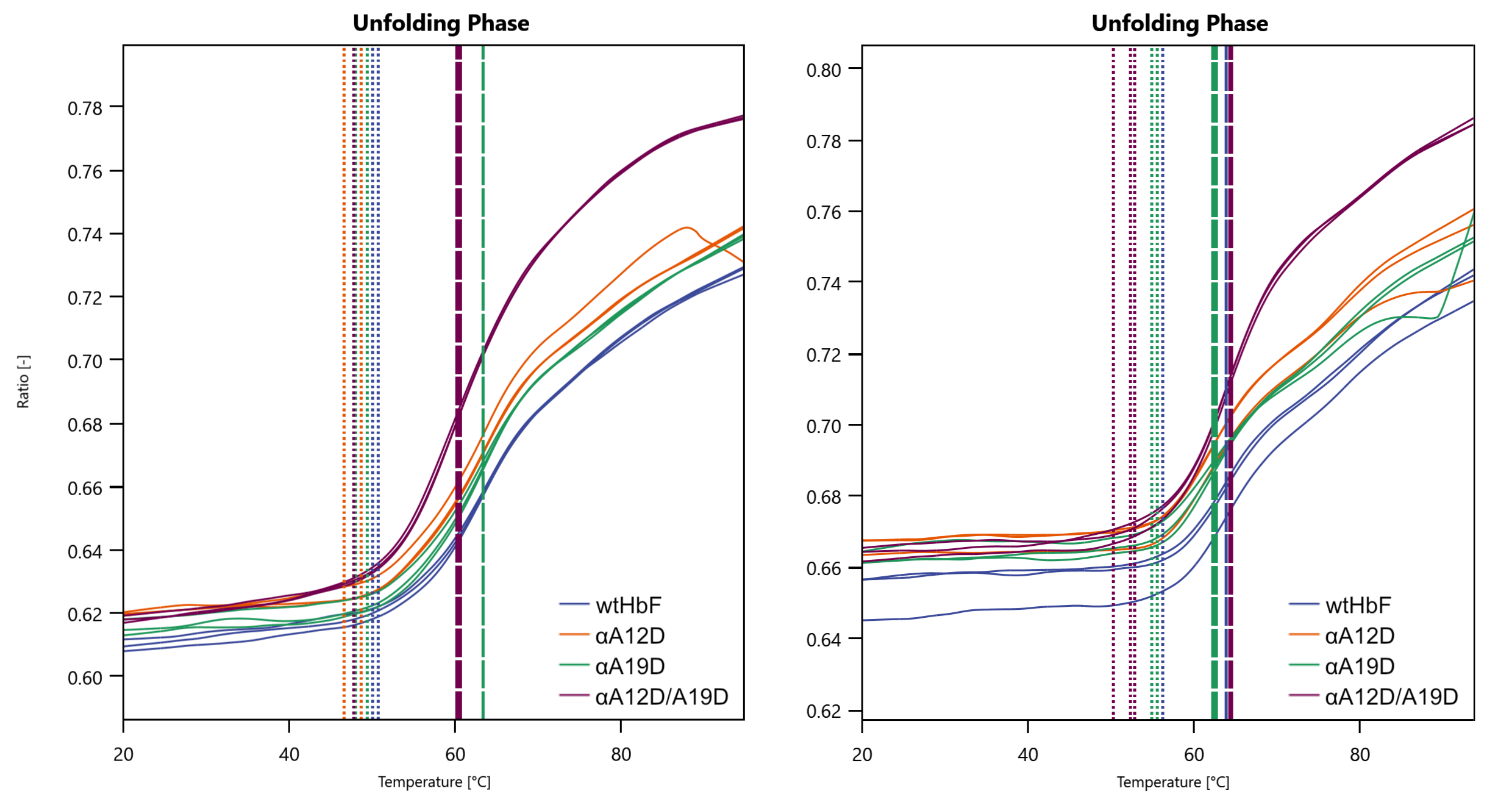


**Figure S3.** Thermal denaturation of wtHbF, αA12D and αA19D, and αA12D/A19D, measured in a Prometheus NT.48 instrument (Nano Temper Technologies). The intrinsic fluorescence at 350:330 nm ratio was followed over a 7 °C/min temperature gradient and onset and transition temperatures was calculated by the PR.therm Control v.2.04 program. Left graph shows the ferric (Fe^3+^) samples, while the right graph shows the O_2_-bound samples.

**Table S1.** nanoDSF thermal denaturation. Independent t-tests (P < 0.05 significance level), bold format indicate significant difference.

| ONSET (Fe^3+^) | wtHbF | αA12D | αA19D | αA12D/A19D |
| --- | --- | --- | --- | --- |
| wtHbF |  | **0.0117** | **0.0379** | **< 0.001** |
| αA12D |  |  | 0.0768 | 0.5478 |
| αA19D |  |  |  | **0.0409** |
| αA12D/A19D |  |  |  |  |
|  | | | | |
| ONSET (Fe^2+^-O_2_) | wtHbF | αA12D | αA19D | αA12D/A19D |
| wtHbF |  | 0.2441 | 0.1415 | **0.0025** |
| αA12D |  |  | 0.4692 | **0.0012** |
| αA19D |  |  |  | **0.0025** |
| αA12D/A19D |  |  |  |  |
|  | | | | |
| INFLECTION (Fe^3+^) | wtHbF | αA12D | αA19D | αA12D/A19D |
| wtHbF |  | 0.4544 | 0.6582 | **< 0.001** |
| αA12D |  |  | 0.5762 | **< 0.001** |
| αA19D |  |  |  | **< 0.001** |
| αA12D/A19D |  |  |  |  |
|  | | | | |
| INFLECTION (Fe^2+^-O_2_) | wtHbF | αA12D | αA19D | αA12D/A19D |
| wtHbF |  | **< 0.001** | **0.0032** | **0.0225** |
| αA12D |  |  | 0.4501 | **< 0.001** |
| αA19D |  |  |  | **< 0.001** |
| αA12D/A19D |  |  |  |  |

# SAXS & modelling


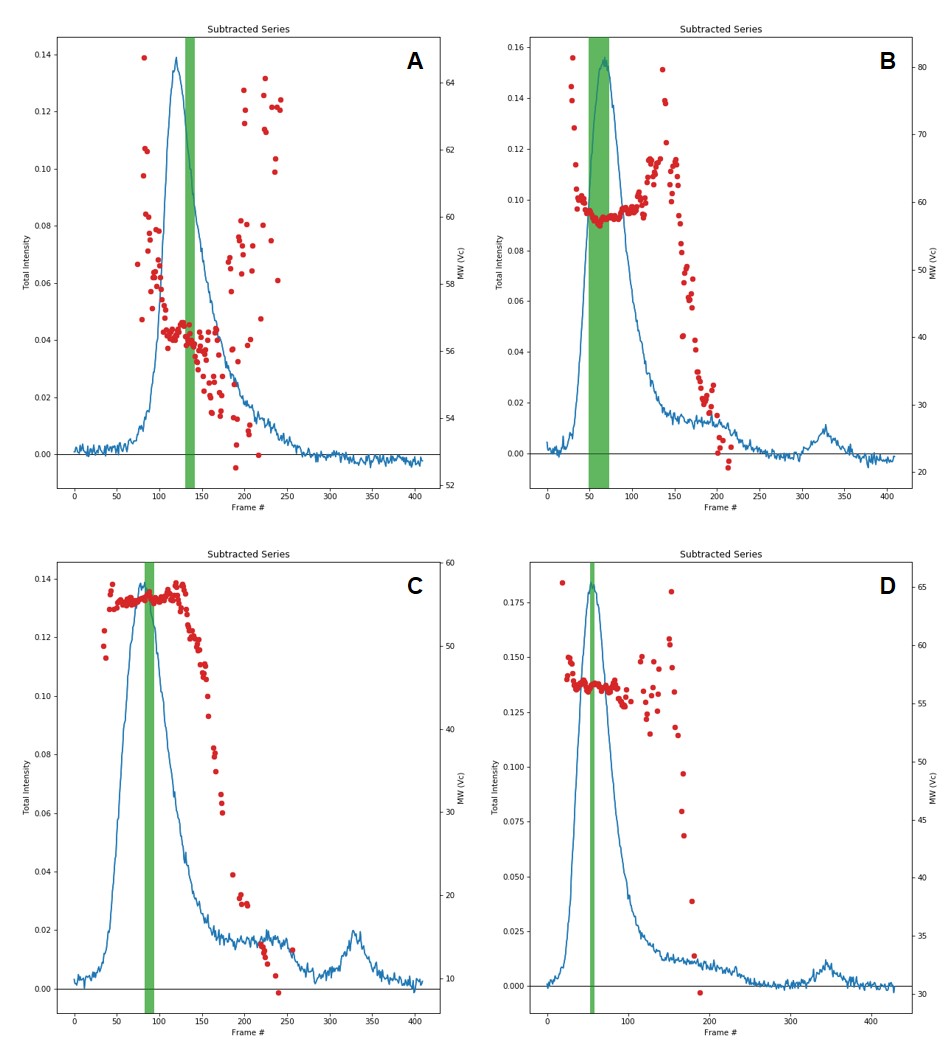


**Figure S4.** Subtracted SEC-SAXS frames. On the left axis the scattering intensity (blue trace), and the right axis an estimate of the MW (red dots). The green band delimit the frames used to create the I(q) for the sample. (**A**) HbF wildtype, (**B**) HbF αA12D, (**C**) HbF αA19D, (**D**) HbF αA12D/A19D.


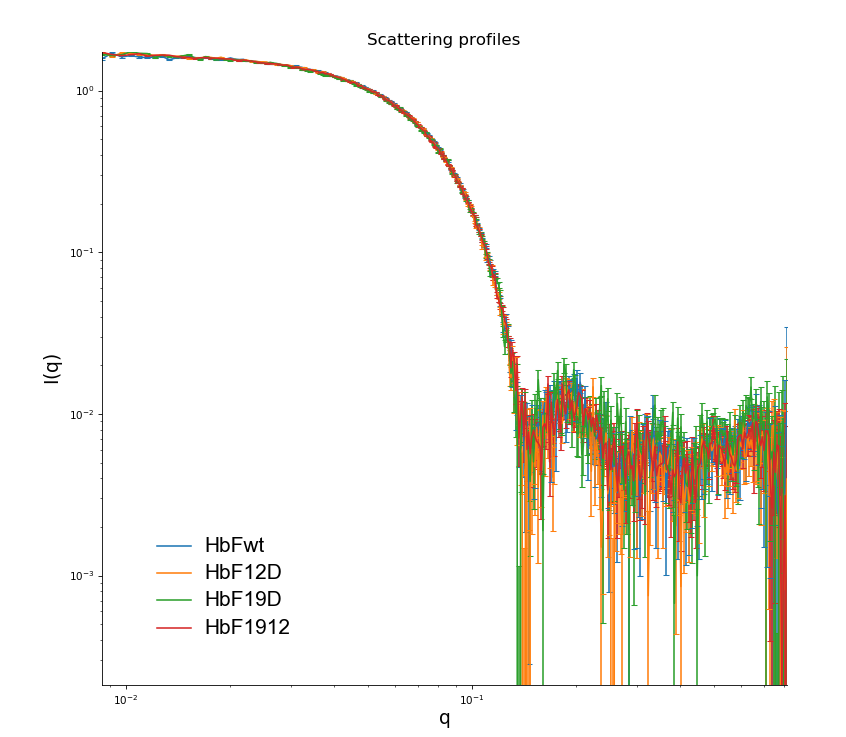


**Figure S5.** Scattering profiles extracted from the SEC-SAXS data.

The SAXS scattering profile is measured in reciprocal distance space, as I(q) where q has units of one over distance (usually 1/Angstrom or 1/nm). We apply a Fourier transform to the data to get information in real space about the macromolecule, as:

$$P\left( r \right)=\frac{r^{2}}{{2\pi}^{2}}\int_{0}^{\infty} q^{2}I(q)\frac{sin(qr)}{qr}dq$$

This produces the P(r) function, also called the pair distance distribution function. The P(r) function contains valuable information about the shape and size of a macromolecule. First, in doing the P(r) function we get an estimate of the maximum dimension of the macromolecule (Dmax). It also provides another, potentially more accurate, way to calculate the R_g_ and I(0). The shape of the P(r) function can also be directly interpreted in terms of the shape of the macromolecule, providing information about the overall shape, such as globular vs. rod-like, or whether the macromolecule contains multiple domains.

Here we estimate the P(r) using a Bayesian approach (IFT) or a regularized inverse Fourier transform (Gnom). The Dmax from the two methods are reported in the last two columns of Table S2.


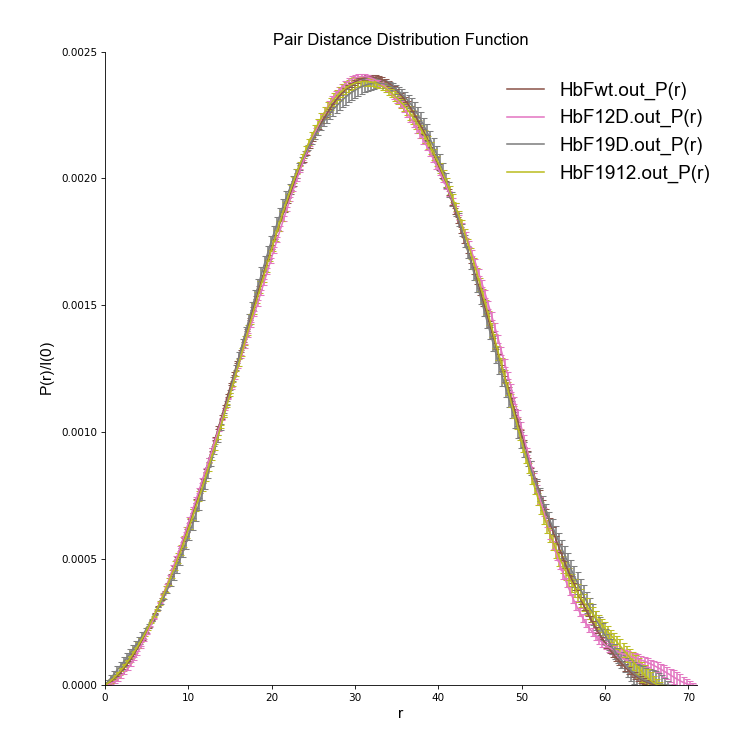


**Figure S6.** Pair distribution profiles.

# Numerical analysis (Franke et al. 2017, Hopkins et al. 2017)

Guinier Analysis

The first two columns in Table S2 are from the Guinier analysis and fitting the following equation to the scattering data at lower scattering vector (q):

$$I(q)\approx I(0)e^{-q^{2}R_{g}^{2}/3}$$

where R_g_ is the radius of gyration and I(0) is the intensity at zero scattering angle (q=0). Because of the exponential in the Guinier approximation, R_g_ and I(0) can be determined by performing a linear fit to a plot of ln(𝐼) vs. 𝑞^2^, called the Guinier plot.

The R_g_ inform about the overall size of the molecule, while I(0) depends on the molecular weight times the concentration. In this case the uncertainty of the concentration meant that the molecular weight is estimated otherwise.

Molecular weight estimation (columns 3 to 6)

SAXS molecular weight calculations are not terribly accurate, a usual rule of thumb is ~10% uncertainty (or more). For this reason, SAXS should not be used to determine the molecular weight of the sample. The main reason to calculate molecular weight from SAXS data is to determine the oligomeric state of the protein in solution.

There are a number of ways to calculate molecular weight from SAXS data:

(1) Molecular weight from absolute scaled I(0)

In our case the scattering intensities are not in absolute scale, so we rule out (1).

(2) Molecular weight by comparing to a reference standard

We did not have a reference standard, so we rule out (2).

(3) Molecular weight from the Porod volume

The estimate from the Porod volume (3) is based on an estimate of the excluded volume of the macromolecule in solution Vp, hence the MW (Vp).

(4) Molecular weight from the volume of correlation (Vc)

The method is based on the experimental observation that Vc^2^/R_g_ is linearly proportional to MW, hence the MW (Vc).

(5) Molecular weight estimation by comparison of scattering to known structures

By finding the nearest structures in shape and size (also the name of the method: Shape & Size), one can obtain estimates for the molecular weight of the sample, hence MW (S&S).

(6) Molecular weight calculation by Bayesian inference from the other molecular weight methods.

A method for calculating a molecular weight using Bayesian inference with the molecular weight calculations from the Porod volume (Vp), volume of correlation (Vc), and comparison to known structures methods (S&S) as the evidence. Statistically this method should be the closest to the MW.

**Table S2.** Scattering results.

| **HbF variant** | **I(0)** | **Rg (Å)** | **MW(Vc)** | **MW(Vp)** | **MW(Bayes)** | **MW(S&S)** | **Dmax Å (IFT)** | **Dmax Å (Gnom)** |
| --- | --- | --- | --- | --- | --- | --- | --- | --- |
| wtHbF | 1.73 +/- 8.82 10^-3^ | 24.21 +/- 0.09 | 56.2 | 60.7 | 58.1 | 61.1 | 62.28 +/- 2.37 | 64 |
| αA12D | 2.39 +/- 3.33e-3 | 24.62 +/- 0.05 | 57.5 | 64.2 | 59.5 | 64.8 | 409.22 +/- 26.7 | 71 |
| αA19D | 2.29 +/- 7.42 10^-3^ | 24.67 +/- 0.13 | 56.4 | 61.7 | 56.9 | 63.4 | 90.37 +/- 5.82 | 68 |
| αA12D/A19D | 3.12 +/- 6.85 10^-3^ | 24.16 +/- 0.09 | 56.6 | 60.5 | 56.9 | 65.3 | 65.88 +/- 2.62 | 67 |

# Rigid body modelling

The tetramer is split in the alpha and gamma chains (unit 1,3 and 2,4 respectively). We used the ATSAS SASREF rigid body modelling software. The four subunits are forced into known contact conditions and the software refines the subunit position to fit the scattering profile.

## Contact condition at 7 Å

Contact conditions

unit 1: alpha, unit 2: gamma, unit 3: alpha, unit 4: gamma

dist 7 Å (example: “distance of 7 Å between unit 1 residue 35 and unit 2 residue 128”)

**1** 35 35 **2** 128 128

**1** 111 111 **2** 115 115

**1** 123 123 **2** 34 34

**3** 35 35 **4** 128 128

**3** 111 111 **4** 115 115

**3** 123 123 **4** 34 34

**1** 96 96 **4** 101 101

**3** 96 96 **2** 101 101


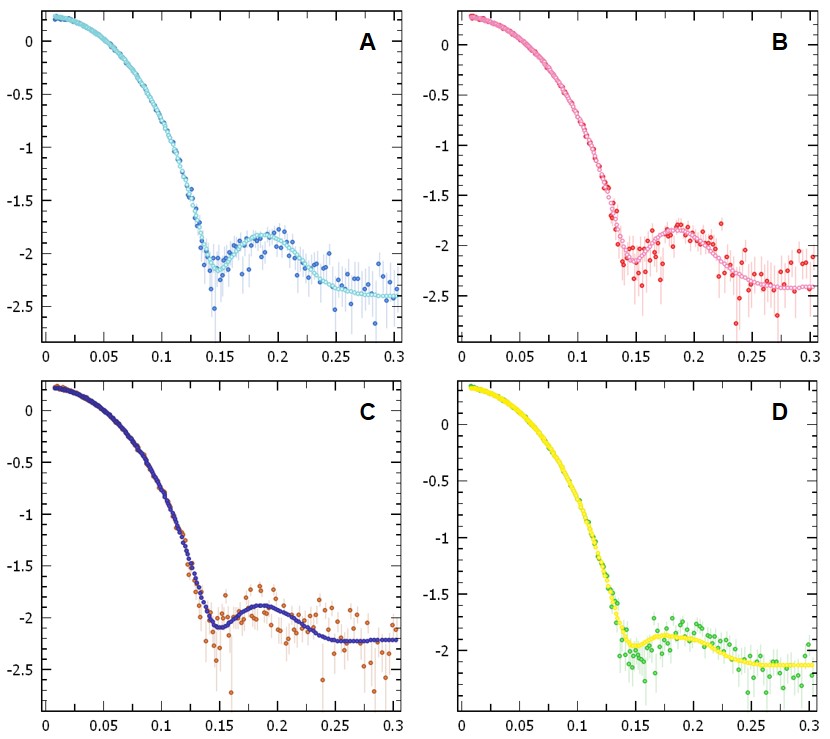


**Figure S7.** Fit of the SASREF docking with 7 Å contact condition. Line is the fit and dots are the scattering data. (**A**) HbF wildtype, (**B**) HbF αA12D, (**C**) HbF αA19D, (**D**) HbF αA12D/A19D.

## Contact condition at 9 Å

Contact conditions

unit 1: alpha, unit 2: gamma, unit 3: alpha, unit 4: gamma

dist 9 Å (example: “distance of 9 Å between unit 1 residue 35 and unit 2 residue 128”)

**1** 35 35 **2** 128 128

**1** 111 111 **2** 115 115

**1** 123 123 **2** 34 34

**3** 35 35 **4** 128 128

**3** 111 111 **4** 115 115

**3** 123 123 **4** 34 34

**1** 96 96 **4** 101 101

**3** 96 96 **2** 101 101


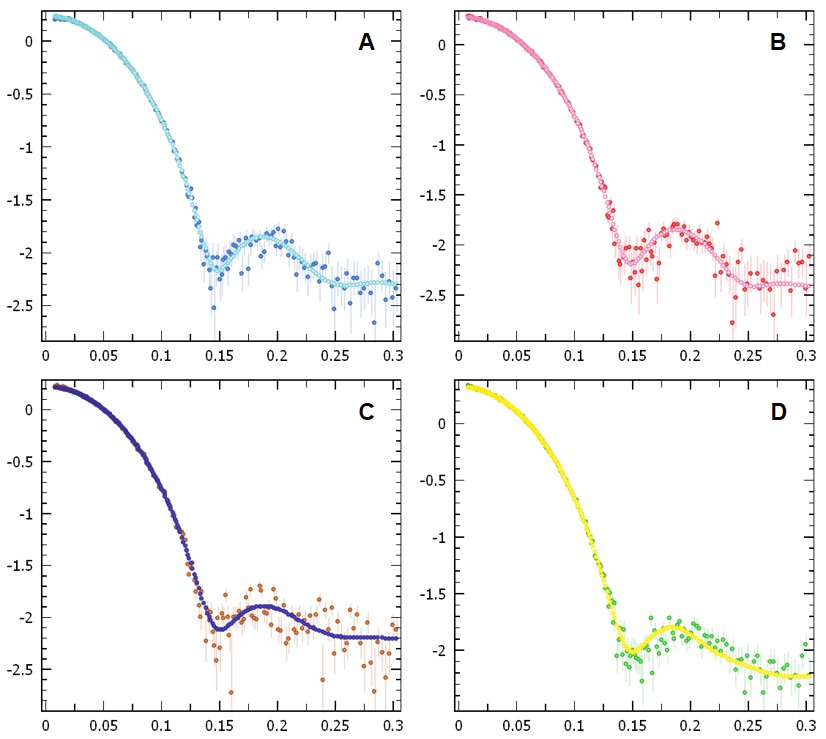


**Figure S8.** Fit of the SASREF docking with 9 Å contact condition. Line is the fit and dots are the scattering data. (**A**) HbF wildtype, (**B**) HbF αA12D, (**C**) HbF αA19D, (**D**) HbF αA12D/A19D.


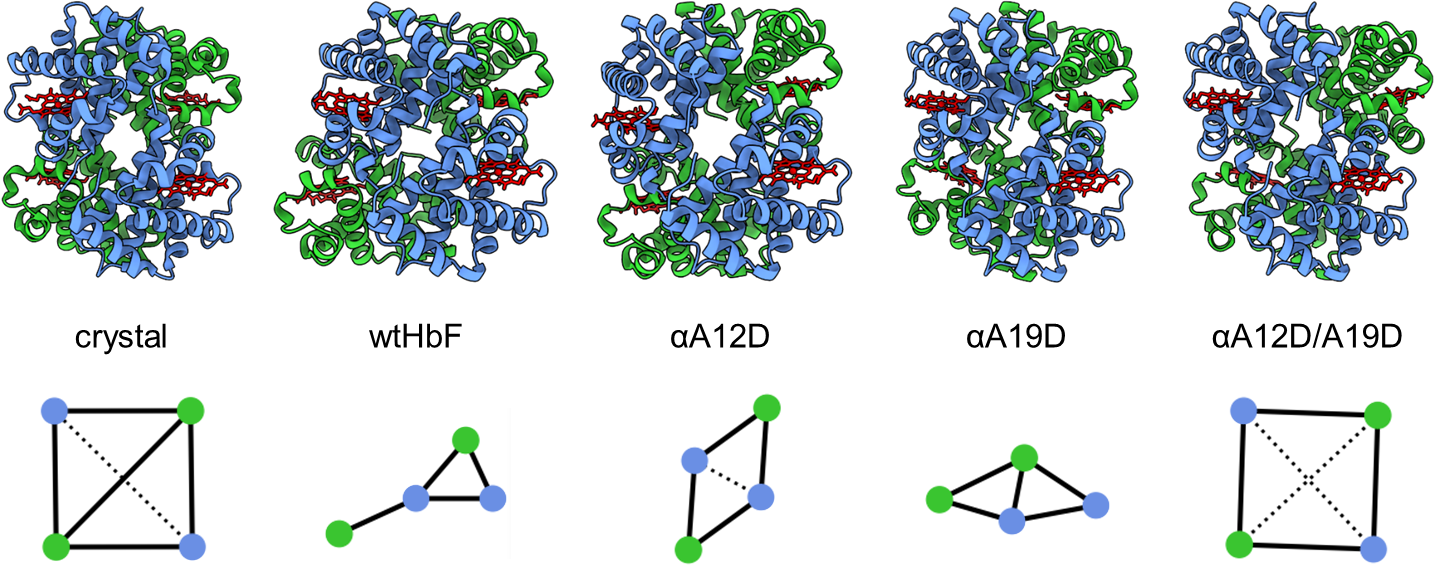


**Figure S9.** The crystal structure of 4MQJ and four 3D models of the recombinant HbFs generated by the rigid-body docking with SASREF, contact condition 9 Å. The α-subunits are displayed in blue and the γ-subunits in green, while the heme groups are red. Below the 3D models are the subunit contact interface diagrams calculated by ChimeraX software for the different structures generated by the rigid body docking. From left to right: crystal structure 4MQJ, in solution SAXS of wtHbF, αA12D, αA19D and αA12D/A19D. Blue dots are the α-chains and green dots are the γ-chains.

Franke, D., M. V. Petoukhov, P. V. Konarev, A. Panjkovich, A. Tuukkanen, H. D. T. Mertens, A. G. Kikhney, N. R. Hajizadeh, J. M. Franklin, C. M. Jeffries, and D. I. Svergun. (2017). ATSAS 2.8: a comprehensive data analysis suite for small-angle scattering from macromolecular solutions. *Journal of Applied Crystallography.* 50 (4)1212-1225. doi: doi:10.1107/S1600576717007786.

Hopkins, J. B., R. E. Gillilan, and S. Skou. (2017). BioXTAS RAW: improvements to a free open-source program for small-angle X-ray scattering data reduction and analysis. *Journal of Applied Crystallography.* 50 (5)1545-1553. doi: doi:10.1107/S1600576717011438.
